# Intergroup relations and dynamics of (dis)integration between youth with immigrant and non-immigrant origins: a scoping review

**DOI:** 10.3389/fpsyg.2025.1681385

**Published:** 2025-11-26

**Authors:** Katja Lötjönen, Hadi Farahani, Jari Martikainen, Marlen Nissinen

**Affiliations:** Department of Social Sciences, Faculty of Social Sciences and Business Studies, University of Eastern Finland, Kuopio, Finland

**Keywords:** educational environments, immigrant, interethnic encounters, intergroup relations, integration, non-immigrant, youth

## Abstract

In light of growing societal diversity, gaining a deeper understanding of intergroup relations is increasingly crucial for fostering inclusive and cohesive communities. This scoping review synthesizes research on immigrant and non-immigrant young people’s perceptions and experiences of intergroup relations and (dis)integration. Analyzing 46 studies conducted between 2010 and 2023 through qualitative content analysis identified that intergroup contact theory and social identity theory are the dominant frameworks with quantitative methods being the most common. Seventeen thematic categories were produced, with the top four being (1) social inclusion or exclusion, (2) cultural or ethnic identity, (3) intergroup contact, and (4) attitudes. While offering insights into young people’s perceptions of interethnic encounters, the review highlights gaps in empirical, methodological, and theoretical diversity. Future research is encouraged to expand existing frameworks, integrate qualitative and mixed-method approaches, and focus more on real-world intergroup and interethnic encounter settings, that potentially enhances understanding of youth meaning-making in diverse ethnic contexts. Focusing on schools as key contexts for intergroup encounters, this review highlights the need to support integrative practices and incorporate cross-cultural after-school programs to enhance educational inclusion.

**Systematic review registration:**

This scoping review is registered on the Open Science Framework (OSF) website: https://osf.io/p2678/overview.

## 1 Introduction

Due to global increases in international migration, societies are becoming more diverse in terms of people’s ethnic and cultural backgrounds. While this development increases opportunities for contact between different ethnic populations (Pettigrew et al., 2010), it may create tensions between them (Crocetti et al., 2021). Therefore, intergroup relations pose a key challenge for contemporary societies regarding how to support cooperation and peaceful coexistence between people from different ethnic backgrounds (Hogg, 2013).

According to Sherif’s (1962) classical definition, intergroup relations refer to “relations between two or more groups and their respective members. Whenever individuals belonging to one group interact, collectively or individually, with another group or its members in terms of their group identifications we have an instance of intergroup behavior” (p. 5). In this definition, intergroup relations are based on people’s self-categorization as being part of a group and interacting with members of other groups based on their group-based social identity. Consequently, intergroup relations can be broadly conceptualized as the ways members of one social group view, think, and feel about; communicate with; and act toward members of another social group. Therefore, intergroup relations examine the perceptions, interactions, and behaviors of individuals and groups in relation to other groups (Hogg, 2013). These dynamics are studied in various ingroup–outgroup contexts such as attitudes, stereotypes, discrimination, prejudices, social identity, and minority–majority relations (see Brown and Gaertner, 2001). Cultural influences and their implications for intergroup relations, including phenomena such as acculturation and integration, often studied within the context of intergroup contact, are of particular interest to intergroup relations researchers focusing on ethnic and cultural groups (see Liebkind, 2001; Triandis and Trafimow, 2001). This scoping review focuses on intergroup relations among immigrant and non-immigrant young people, placing specific focus on (dis)integration.

An extensive body of research on intergroup relations in the context of migration has used the integration framework (e.g., Figgou and Baka, 2018). Migrant integration has been examined at different levels, ranging from politics and the economy to the everyday encounters between people (see, e.g., Hainmueller et al., 2016; Heath and Schneider, 2021). Due to its many dimensions, migrants’ integration into diverse aspects of host societies is a multifaceted and long-term process, whereby migrants’ prior experiences and histories as well as social and societal contexts of the host society are set into dialogue (Ishiyama and Silva, 2020; Hur, 2023). Recently, approaches based on integration have been found to be problematic because instead of mutual adaptations between different groups of people, they mostly focus on one-way migrants’ adaptation to host societies. In this scoping review, we understand integration as an interactive process, which “depends as much on the newcomers’ aspirations to belong as it does on the host’s intent to include” (Hur, 2023, p. 3248). In this approach, integration appears as negotiating belonging (Hur, 2023), in which both immigrant and non-immigrant populations are involved.

In his contact hypothesis, Allport (1954) argued that intergroup contact reduces prejudice when four optimal conditions are met: the groups cooperate, and they have equal status, a common goal, and institutional support. Aiming to increase integration, these principles could be exemplified for instance in educational settings through practices such as collaborative group work with balanced roles, joint multicultural community projects, and school policies that actively promote inclusion and intercultural competence. Moreover, Pettigrew (1998) extended Allport’s (1954) contact hypothesis by emphasizing personal self-disclosure and outgroup friendships as key mechanisms for improving intergroup relations, which are particularly relevant among youth. In their meta-analysis, Pettigrew and Tropp (2006) posited that intergroup contact may have a positive impact on outgroup evaluations even if Allport’s (1954) optimal conditions are not met. Hence, there is a widely shared consensus that positive intergroup contact contributes to more positive emotions toward and perceptions of ethnic outgroups and improves intergroup attitudes (Brown and Hewstone, 2005; Pettigrew and Tropp, 2006; Kotzur et al., 2019). In contrast, anxiety, threat, and uncertainty related to another group of people reduce willingness to engage with them (Islam and Hewstone, 1993; Scuzzarello, 2012), and negative intergroup contacts may lead to adverse intergroup outcomes (Pettigrew, 1998; Pettigrew et al., 2010).

While intergroup contact encompass both positive and negative experiences—ranging from cooperation and mutual understanding to tension and conflict (Dovidio et al., 2011)—it is outcomes are not uniformly integrative. The meanings constructed through such encounters can both promote and challenge integration, reflecting how contact can simultaneously foster cohesion and reinforce division. In this sense, intergroup relations represent a dynamic field in which integration and disintegration unfold in parallel, shaped by multiple interacting factors. The permeability of group boundaries, for instance, affects how groups perceive and relate to one another, as more fluid boundaries tend to enable inclusion and cooperation, while rigid ones can sustain exclusion and conflict (Armenta et al., 2017). Similarly, ideological orientations and identity processes influence how intergroup experiences are interpreted, as dominance-oriented worldviews and perceived threats can reproduce prejudice and separation (Duckitt et al., 2002; Dawd et al., 2021). In line with this, prior research has examined not only positively valence processes, such as inclusion (e.g., Dovidio et al., 2005; van Prooijen et al., 2004) which promote integration, but also problematic processes, such as exclusion (e.g., Dovidio et al., 2005), discrimination (e.g., Dixon et al., 2010; Schaeffer and Kas, 2024), and segregation (e.g., Dixon and Durrheim, 2003; Durrheim and Dixon, 2005). Drawing on this body of work, we understand inclusion, exclusion, discrimination, and segregation as interrelated processes that constitute the dynamics of (dis)integration.

Intergroup relations between members of different ethnic and cultural groups (e.g., migrant and host national groups) are particularly significant among adolescents because during that phase of their lives, young people broaden their circle of life towards their peers (Karataş et al., 2023). Although several factors, such as parental and peer norms, influence adolescents’ intergroup attitudes and willingness to engage in intergroup contact (Jasinskaja-Lahti et al., 2011), young people build their intergroup attitudes based on their everyday contacts with peers coming from different ethnic backgrounds (Pettigrew, 1998; Elias et al., 2022; Karataş et al., 2023). These contacts can take place during leisure activities (Kim, 2012) and especially at schools (Bohman and Miklikowska, 2021). For example prior studies have shown racialized representations significantly contribute to school exclusion by shaping institutional practices and affecting students’ self-perceptions (Howarth, 2004; see also Zion et al., 2017). However, students also resist and challenge these oppressive dynamics within schools (Howarth, 2004), e.g., through disengagement in the critical discussions at schools (Zion et al., 2017), resisting the racial stereotypes to prove them wrong, or to resist schools policies by questioning them (Simpson Bueker, 2017).

Despite extensive research on adolescents’ intergroup relations, less is known about how these dynamics are perceived and experienced by young people in their everyday lives, particularly across immigrant and non-immigrant groups. In this scoping review, we therefore focus on (dis)integration dynamics as perceived by young people with immigrant and non-immigrant origins, examining how their day-to-day intergroup encounters shape experiences of inclusion, exclusion, and related (dis)integration processes.

## 2 Aim of the review

This scoping review aims to systematically map and explore the existing research on perceptions and experiences of intergroup relations among young people from different ethnic backgrounds, with a particular focus on everyday (dis)integration. This encompasses the daily behaviors and practices that occur among laypeople in their routine interactions or perceptions they have of actual or potential encounters with people from other cultural origins. We conceptualize these dynamics, such as discrimination, exclusion, inclusion, segregation, and acculturation, as integral to the everyday (dis)integration of various cultural groups, potentially facilitating or hindering the integration process. Our population context consists of young people of both immigrant and non-immigrant origins. We employ intergroup relations as an overarching framework to guide our analysis and to structure the presentation and discussion of our findings. Through our comprehensive review of the existing literature, our review aims to identify gaps in the current literature and suggest potential areas for future research. To the best of our knowledge, there are no prior scoping reviews focusing on this specific topic.

## 3 Methods

During the process of this scoping review, relevant key concepts, primary sources, types of available evidence, and theoretical frameworks used in previous research on immigrants’ and native youths’ intergroup relations were identified (Arksey and O’Malley, 2005). A search protocol was developed *a priori* for this scoping review based on the Joanna Briggs Institute methods manual for scoping reviews (Aromataris et al., 2024). Findings were reported in accordance with the checklist provided by the preferred reporting items for systematic reviews and meta-analyses extension for scoping reviews (PRISMA-ScR; Tricco et al., 2018). The scoping review protocol was registered on the Open Science Framework and is accessible through the following link: https://doi.org/10.17605/OSF.IO/P2678. Finally, this review assesses the quality of the studies conducted, synthesizes the main findings from the existing literature, and addresses knowledge gaps for future research (Munn et al., 2018).

### 3.1 Search strategy and identification of relevant studies

We conducted an extensive literature search for peer-reviewed articles published between 2010 and 2023 using the following databases: Web of Science, Scopus, PubMed, ScienceDirect (Elsevier), Sage, SocINDEX, PsycINFO, JSTOR, and ProQuest. To develop the search strategy, a preliminary search of prior literature was conducted. Based on this review, a search strategy was formulated using a combination of complete and truncated keywords. The search was conducted between November and December 2023 using the following search terms:

“inter* **OR** group* **OR** social* **OR** relation* **OR** encounter* **OR** belonging*

**AND** local* **OR** school*

**AND** immigrant* **OR** Ukrainian* **OR** Finnish* **OR** “Middle East” **OR** Afghan* **OR** Arab* **OR** Syria* **OR** Iraq* **OR** Finland* **OR** Ukraine*

**AND** teen* **OR** youth* **OR** young* **OR** juvenile* **OR** pupil* **OR** child* **OR** adolescent* **OR** refugee* **OR** “asylum seeker” **OR** stateless* **OR** undocumented* **OR** “illegal migrant”

**AND** perception* **OR** perspective* **OR** understanding* **OR** insight* **OR** interpretation* **OR** impression*

**AND** integration* **OR** exclusion* **OR** inclusion* **OR** segregation* **OR** discrimination* **OR** acculturation*”.

This scoping review is part of a larger research project carried out in the context of Finnish society. Although search terms such as “Finland,” “Ukraine,” and “Middle East” were included to capture studies most relevant to the project population, broader general keywords on intergroup relations, youth, and integration were also used to minimize potential geographic bias and ensure the search could capture relevant literature from other contexts. Other search terms, including “exclusion,” “inclusion,” “segregation,” “discrimination,” and “acculturation,” were selected based on previous research framing integration as a dynamic process encompassing both positive and negative aspects (see, e.g., Dixon et al., 2010; Dovidio et al., 2005; Durrheim and Dixon, 2005; van Prooijen et al., 2004). These terms were chosen to reflect the complexity of intergroup relations, capturing the various processes that either promote or hinder integration.

### 3.2 Selection of sources of evidence and eligibility criteria

After the database search, the outcomes were bulk imported to the Covidence^1^ platform. Covidence automatically removed duplicates upon import. Sources were selected and passed the title and abstract screening phase based on the inclusion criteria if they (1) assessed intergroup relations between host populations and immigrants, refugees, asylum seekers, or other ethnic groups among at least two different ethnic or cultural groups; (2) focused on youths ages 13–30^2^; and (3) were published in English. There were considerable diversity and inconsistency in defining the age range of youth, adolescents, or young people, terms often used interchangeably. For instance, in social policy, the European Union defines these ranges as 15–29, 13–30, or 18–30, depending on the context (Council of Europe, 2019). Developmentally, the World Health Organization (2025) sets the range at 10–19, while psychological models, such as Erikson’s psychosocial model, define it as 12–18 (Erikson, 1993). Considering these, our review adopted a broad age range of 13–30 years to capture both adolescence and emerging adulthood (18–29), reflecting the diversity of age groups represented in the included studies and acknowledging that the transition to adulthood often begins earlier and extends longer for many young people (Arnett, 2004).

**Figure 1:**
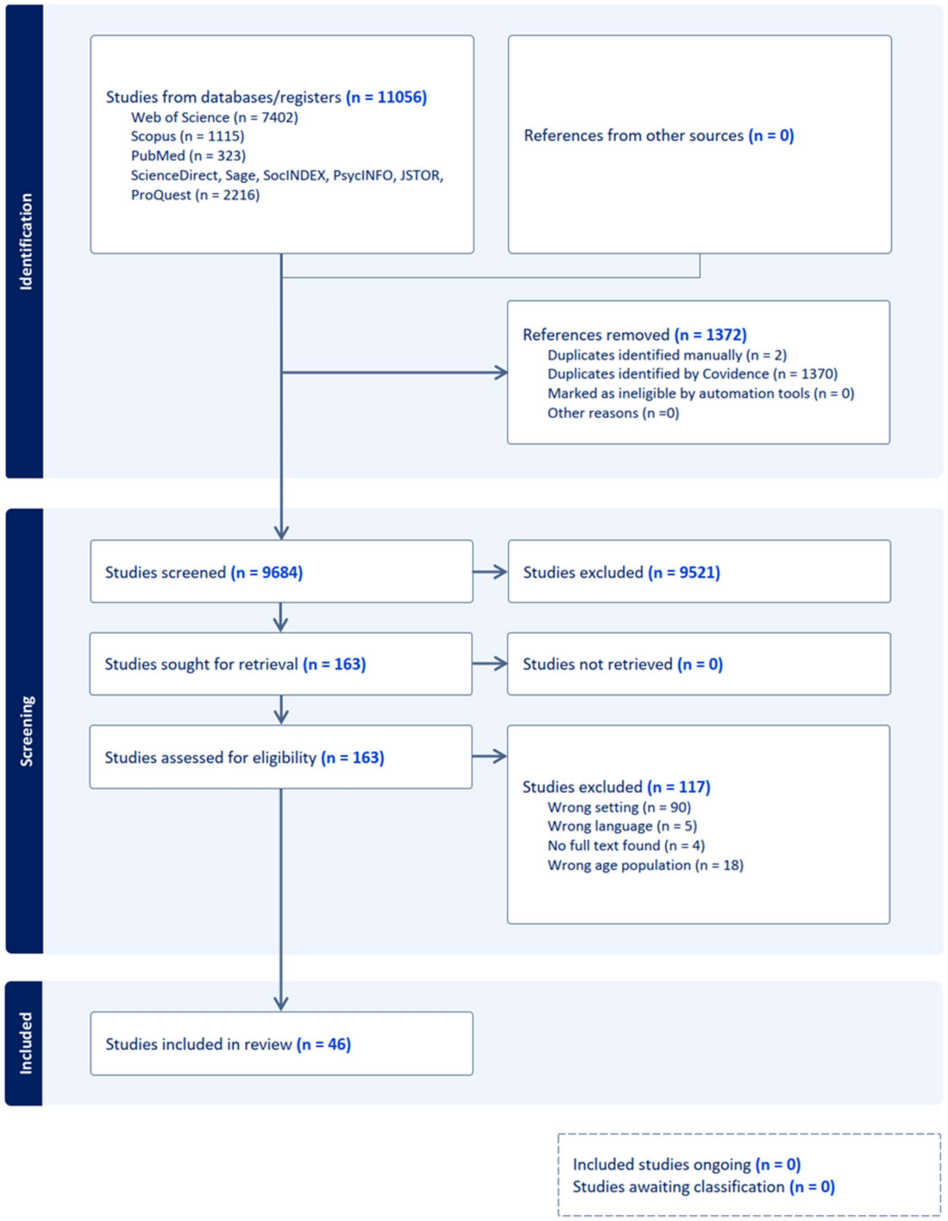
PRISMA flowchart of data charting process.

Articles were excluded if they were published in languages other than English, were not peer-reviewed, or did not include participants within the targeted age range. Many excluded records were also non-empirical sources, such as conference proceedings, theses, dissertations, gray literature, or newspaper reports. Once all conditions were satisfied, the articles advanced to the full-text review phase. During this stage, the articles underwent thorough reading to verify compliance with the inclusion and exclusion criteria. During these phases, two authors (Hadi Farahani and Katja Lötjönen) independently conducted the assessments. In cases of disagreement, a third author (Marlen Nissinen) was consulted to reach a consensus. Articles that were selected then advanced to the data extraction phase. The search strategy resulted in 46 studies being included in this scoping review (Figure 1).

### 3.3 Data charting process and data items

Articles meeting the eligibility criteria after the full text review phase advanced to the data extraction phase. Three authors (Hadi Farahani, Katja Lötjönen, and Marlen Nissinen) independently reviewed the selected articles and extracted relevant information into a data extraction sheet. The data extraction sheet included information on authorship, year of publication, the country of the study, aims, contexts of assessing intergroup relations (the specific dimensions or aspects of intergroup dynamics that the studies focused on), population contexts (detailed characteristics of the study sample and the direction of assessed phenomena in the context of intergroup situations), theoretical frameworks, method (quantitative, qualitative, mixed methods, review), number of participants, and main findings (Supplementary material). The extracted data from each reviewer were then compared and finalized through consensus, ensuring the accuracy of the information.

### 3.4 Critical appraisal of individual sources of evidence

The quality and reliability of the included studies were evaluated in this scoping review using the relevant JBI critical appraisal checklists (Aromataris et al., 2024). The JBI tools assess studies across several methodological dimensions using categorical ratings of Yes, No, Unclear, or Not applicable. The appraisal results were used in two ways: first, to identify common methodological strengths and weaknesses and assess the overall robustness of the evidence; and second, to inform the discussion on research gaps and directions for future studies. The 46 included studies comprised qualitative, quantitative (including experimental), and mixed-methods research, as well as one review and a book chapter summarizing two studies^3^. Each study was independently appraised by three authors (Hadi Farahani, Katja Lötjönen, and Marlen Nissinen), with discrepancies resolved through discussion and consensus. Detailed results of the appraisal are presented in Supplementary material.

### 3.5 Synthesis of empirical results

We used qualitative content analysis to process and summarize the findings of the included studies. This method involves classifying data using units of analysis that range from individual words to broader linguistic expressions (Schreier, 2012). We extracted the main findings from each reviewed article and organized them into thematic categories based on the primary aspect of intergroup relations explicitly addressed in the results. For example, if an article’s findings focused predominantly on attitudes, it was assigned to the thematic category Attitudes. To ensure reliability and consistency, one researcher conducted the initial coding, which was then reviewed and discussed with other team members. This collaborative process helped maintain coherence across themes and reduced the potential for individual bias.

## 4 Results

### 4.1 Population context

The population contexts of the studies differed from each other in terms of the types of ethnic groups and from whose perspective the studies were conducted: host population group/majority group perspective, immigrant group/minority group perspective, or both perspectives.

We identified three population contexts based on the types of ethnic groups that were studied or concepts used to refer to these groups in included articles. First, most studies focused on the relations between host populations and immigrant (i.e., refugee or asylum seeker) populations, which in many cases overlapped with majority (host populations) and minority (immigrant populations) groups. Second, some studies focused on the majority–minority setting using these category terms systematically throughout the study. Third, a number of studies included diverse ethnic groups or concepts referring to them. Finally, one study (Priest et al., 2014) was a systematic review, which we did not include in our population context categorization.

A total of 24 studies explored intergroup relations between adolescents of the host country and another group, such as immigrants or refugees. Most of these studies were done in Europe, most commonly, Germany (Brenick et al., 2012; Beißert et al., 2020; Aral et al., 2022; Beißert and Mulvey, 2022) and Turkey (Çelebi et al., 2014; Gönültaş and Mulvey, 2022, 2023a,b). Furthermore, two studies in this population context were conducted in the U.S. (Dryden-Peterson, 2010; Hitti and Killen, 2023), one in Canada (Kaufmann, 2021), and one in Chile (Chávez et al., 2021). Out of the 24 studies, nine approached intergroup relations from the host country adolescents’ perspective (Beißert et al., 2020; Aral et al., 2022; Beißert and Mulvey, 2022; Gönültaş and Mulvey, 2022, 2023a, 2023b; Yüksel et al., 2022; Palmer et al., 2023; R’boul et al., 2023), thirteen from both host and immigrant adolescents’ perspective (Dryden-Peterson, 2010; Brenick et al., 2012; Jumageldinov, 2014; Çelebi et al., 2014; Plenty and Jonsson, 2017; van Bergen et al., 2017; Belet, 2018; Mazzone et al., 2018; Chávez et al., 2021; Hooijsma and Juvonen, 2021; Kaufmann, 2021; Carter-Thuillier et al., 2023; Lintner et al., 2023), and one from immigrant adolescents’ perspective (Korem and Horenczyk, 2015). Additionally, Spiel and Strohmeier (2012) combined diverse population settings in their book chapter on peer relations.

In addition, 10 studies were built on majority and minority setting. Five of them were conducted from the majority group perspective (Banfield and Dovidio, 2013; Bikmen and Sunar, 2013; Stark et al., 2015; Bohman and Miklikowska, 2021; Maor and Gross, 2023), one from the minority group perspective (Munayer and Horenczyk, 2014), and the remaining four studies combined both majority and minority group perspectives (Kisfalusi et al., 2020; Wang et al., 2020; Kretschmer and Leszczensky, 2022; Spiegler et al., 2024). Apart from one study conducted in the U.S. (Banfield and Dovidio, 2013) and another in China (Wang et al., 2020), all other majority and minority setting studies were carried out in the European context.

Finally, we identified 11 studies where population contexts were more diverse (Rienties and Nolan, 2014; Hitti and Killen, 2015, 2023; Brenick and Romano, 2016; Szabó et al., 2020; Smith and Minescu, 2021; Saunders et al., 2022; Zhou et al., 2022; Castro et al., 2023; Hitti et al., 2023; Piipponen, 2023). Seven of these studies were conducted in the North American context (Hitti and Killen, 2015, 2023; Brenick and Romano, 2016; Saunders et al., 2022; Zhou et al., 2022; Castro et al., 2023; Hitti et al., 2023) and four in the European context (Rienties and Nolan, 2014; Szabó et al., 2020; Smith and Minescu, 2021; Piipponen, 2023). Differing from the two previous context settings, these studies did not focus on perceptions between host/majority and immigrant/minority groups. Rather, the population context was more diverse. For example, Castro et al. (2023) studied several immigrant groups and assessed how high school students belonging to these groups developed a sense of connection through an intergroup dialogue program. Hitti and Killen (2023) studied inclusive and exclusive group norms in three different host population/majority vs. immigrant/minority groups from multiple perspectives: from the perspective of the host population/majority (non-Arab Americans), the host population/majority and immigrants/minority (non-Asian and Asian Americas), and immigrants/minority (Lebanese). As a final example, Rienties and Nolan (2014) studied international students’ perspective of how they built and maintained relations with co-nationals, multi-nationals, and host-national students in the UK.

To summarize the results on population contexts in our scoping review, intergroup relations between different ethnic groups among adolescents were most often studied from host/majority perspectives or combining host/majority and immigrant/minority perspectives. Only two studies examined intergroup relations focusing solely on minority/immigrant/refugee perspectives. Furthermore, while host/majority and immigrant/minority contexts were used more in European countries, diverse contexts were used more in the U.S.

### 4.2 Theories used in reviewed articles

The most frequently utilized theoretical framework among the included studies was intergroup contact theory, originally proposed by Allport (1954), which was used in 11 articles (Bikmen and Sunar, 2013; Stark et al., 2015; Brenick and Romano, 2016; Wang et al., 2020; Bohman and Miklikowska, 2021; Hooijsma and Juvonen, 2021; Kaufmann, 2021; Smith and Minescu, 2021; Gönültaş and Mulvey, 2022; Zhou et al., 2022; Castro et al., 2023). In addition, later theoretical developments of the contact theory were utilized in three studies: interethnic contact theory (Belet, 2018), equal status contact theory (Dryden-Peterson, 2010), and intergroup contact model (Hitti and Killen, 2023).

The second most utilized theoretical framework was social identity theory (SIT), applied in seven studies (Jumageldinov, 2014; Brenick and Romano, 2016; Plenty and Jonsson, 2017; Kisfalusi et al., 2020; Hooijsma and Juvonen, 2021; Smith and Minescu, 2021; Maor and Gross, 2023). Similarly, seven studies were based on social reasoning development (SRD), which combines premises of SIT and social domain theory (Beißert et al., 2020; Beißert and Mulvey, 2022; Yüksel et al., 2022; Gönültaş and Mulvey, 2023a; Hitti and Killen, 2023; Hitti et al., 2023; Palmer et al., 2023).

Other theoretical frameworks used in the included studies were social cognitive theory (van Bergen et al., 2017; Chávez et al., 2021; Gönültaş and Mulvey, 2022), acculturation approaches (Munayer and Horenczyk, 2014; Szabó et al., 2020; R’boul et al., 2023), the socio-ecological approach (Priest et al., 2014; Mazzone et al., 2018), social capital theory (Dryden-Peterson, 2010; Rienties and Nolan, 2014), network theories (Rienties and Nolan, 2014; Lintner et al., 2023), and social dominance theory (Wang et al., 2020; Maor and Gross, 2023). Additionally, four studies did not refer to any particular theoretical framework but based their approach on a literature review related to the topic of the study (Banfield and Dovidio, 2013; Korem and Horenczyk, 2015; Kretschmer and Leszczensky, 2022; Gönültaş and Mulvey, 2023b), while the remaining 12 studies drew on various other theoretical frameworks beyond those mentioned above (Brenick et al., 2012; Spiel and Strohmeier, 2012; Çelebi et al., 2014; Hitti and Killen, 2015; van Bergen et al., 2017; Szabó et al., 2020; Aral et al., 2022; Saunders et al., 2022; Carter-Thuillier et al., 2023; Palmer et al., 2023; Piipponen, 2023; Spiegler et al., 2024). The theoretical frameworks employed in the included articles were instrumental in conceptualizing and enhancing the understanding of various cognitive aspects of group-based divisions among individuals from diverse cultural backgrounds. Consequently, approaches that emphasize socio-cognitive processes were predominantly utilized.

### 4.3 Methods used in reviewed articles

When considering the methodology of the reviewed studies, 34 used quantitative research methods (Brenick et al., 2012; Banfield and Dovidio, 2013; Bikmen and Sunar, 2013; Munayer and Horenczyk, 2014; Rienties and Nolan, 2014; Çelebi et al., 2014; Hitti and Killen, 2015, 2023; Stark et al., 2015; Brenick and Romano, 2016; Plenty and Jonsson, 2017; Belet, 2018; Beißert et al., 2020; Kisfalusi et al., 2020; Szabó et al., 2020; Wang et al., 2020; Bohman and Miklikowska, 2021; Chávez et al., 2021; Hooijsma and Juvonen, 2021; Kaufmann, 2021; Smith and Minescu, 2021; Aral et al., 2022; Beißert and Mulvey, 2022; Gönültaş and Mulvey, 2022, 2023a, 2023b; Kretschmer and Leszczensky, 2022; Yüksel et al., 2022; Zhou et al., 2022; Hitti et al., 2023; Lintner et al., 2023; Maor and Gross, 2023; Palmer et al., 2023; Spiegler et al., 2024). Specifically, four utilized experimental methods (Banfield and Dovidio, 2013; Belet, 2018; Beißert et al., 2020; Yüksel et al., 2022), nine were qualitative studies (Dryden-Peterson, 2010; Korem and Horenczyk, 2015; van Bergen et al., 2017; Mazzone et al., 2018; Saunders et al., 2022; Carter-Thuillier et al., 2023; Castro et al., 2023; Piipponen, 2023; R’boul et al., 2023), one used mixed methods (Jumageldinov, 2014), one was a review study (Priest et al., 2014), and one was a book chapter (Spiel and Strohmeier, 2012). The extensive use of quantitative methodology in this area of research means that the studies typically used large sample sizes and questionnaires, aiming for the generalizability of results.

Regarding the study design utilized in the included articles, 26 used a cross-sectional design (Brenick et al., 2012; Bikmen and Sunar, 2013; Jumageldinov, 2014; Munayer and Horenczyk, 2014; Çelebi et al., 2014; Hitti and Killen, 2015, 2023; Stark et al., 2015; Brenick and Romano, 2016; Plenty and Jonsson, 2017; Beißert et al., 2020; Kisfalusi et al., 2020; Szabó et al., 2020; Wang et al., 2020; Hooijsma and Juvonen, 2021; Kaufmann, 2021; Aral et al., 2022; Beißert and Mulvey, 2022; Gönültaş and Mulvey, 2022, 2023a; Zhou et al., 2022; Carter-Thuillier et al., 2023; Hitti et al., 2023; Lintner et al., 2023; Maor and Gross, 2023; Palmer et al., 2023). In addition, five utilized a longitudinal study design (Rienties and Nolan, 2014; Stark et al., 2015; Bohman and Miklikowska, 2021; Chávez et al., 2021; Kretschmer and Leszczensky, 2022). The cross-sectional design typically utilized in the included research constrains the ability to evaluate the consistency of results and establish cause-and-effect relationships of the findings.

## 5 Empirical findings

**Figure 2:**
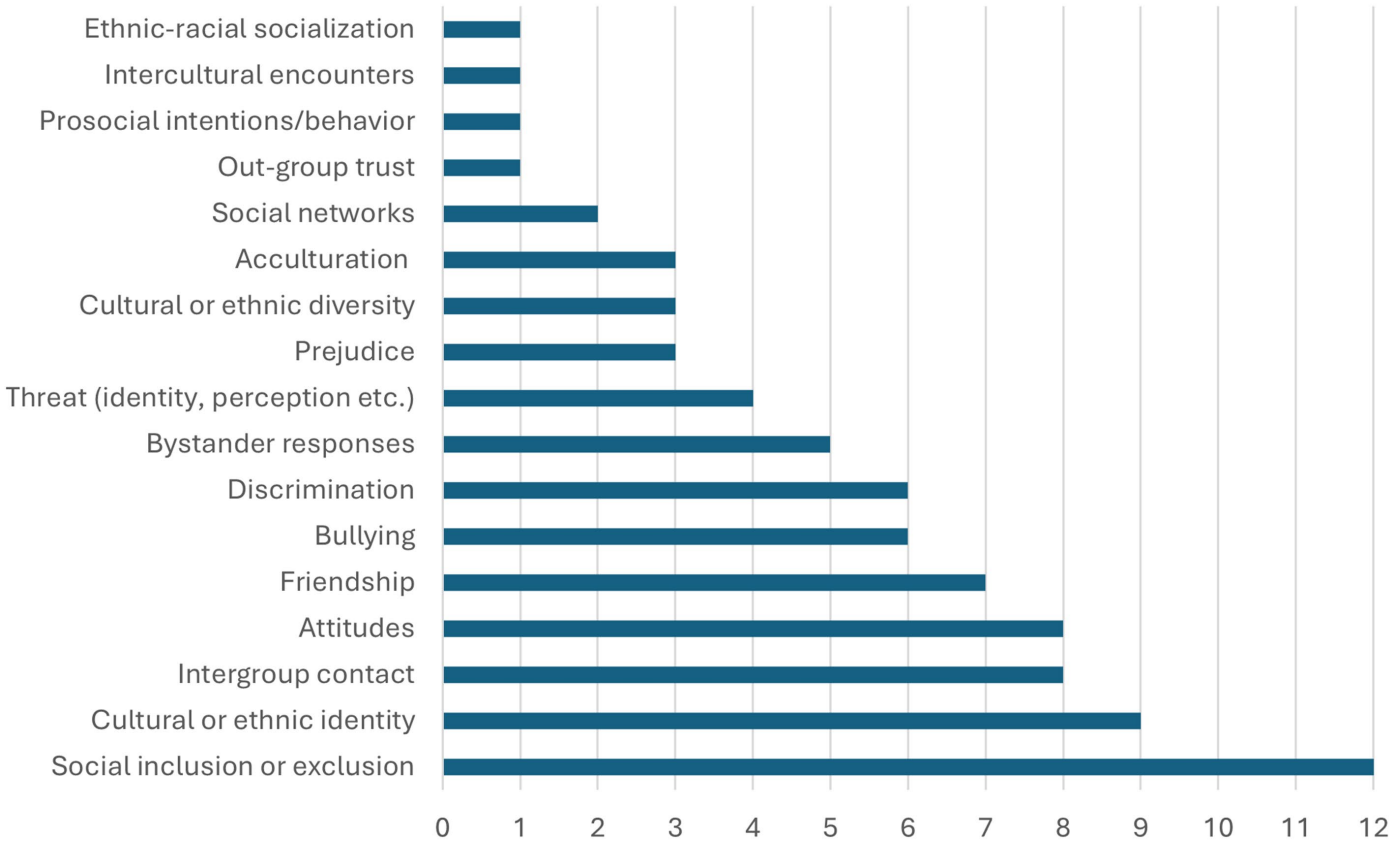
The number of studies for each thematic category.

We analyzed the main findings of the 46 studies focusing on intergroup relations included in this review using content analysis. From this dataset, we created a total of 17 thematic categories (see Figure 2) clustering different aspects of intergroup relations related to the (dis)integration of various ethnic groups of young people.

Next, we provide a synthesis of the key findings, concentrating on the four most common thematic categories: (1) social inclusion or exclusion, (2) cultural or ethnic identity, (3) intergroup contact, and (4) attitudes. As a rule, each study focused on at least one thematic category. However, six studies combined two or more thematic categories indicating the intertwined and multifaceted connections between intergroup relations and integration: Carter-Thuillier et al. (2023) and Brenick and Romano (2016) used social inclusion or exclusion and cultural or ethnic identity; Bikmen and Sunar (2013) used cultural or ethnic identity, intergroup contact, and attitudes; Chávez et al. (2021) used intergroup contact and attitudes; Maor and Gross (2023) used social inclusion or exclusion and attitudes; and Hooijsma and Juvonen (2021) used intergroup contact and attitudes. In the following four subsections, we first provide a detailed description of the findings and subsequently identify the main patterns that emerge from them.

### 5.1 Social inclusion or exclusion

A total of 12 studies examined social inclusion and exclusion, making it the most common thematic category (Hitti and Killen, 2015, 2023; Brenick and Romano, 2016; Plenty and Jonsson, 2017; Beißert et al., 2020; Beißert and Mulvey, 2022; Kretschmer and Leszczensky, 2022; Yüksel et al., 2022; Carter-Thuillier et al., 2023; Lintner et al., 2023; Maor and Gross, 2023; Palmer et al., 2023). The most typically employed theoretical frameworks were social reasoning development (Hitti and Killen, 2023; Beißert et al., 2020; Beißert and Mulvey, 2022; Yüksel et al., 2022; Palmer et al., 2023) and social identity theory (Maor and Gross, 2023; Plenty and Jonsson, 2017). Besides of one qualitative study (Carter-Thuillier et al., 2023), all other utilized quantitative methods. The findings of the studies elaborated different conditions and factors contributing to social inclusion or exclusion in intergroup relations among young people belonging to different ethnic groups.

Beißert et al. (2020) aimed to understand German adolescents’ (ages 10–17) perceptions of Syrian refugee adolescents’ integration in Germany. They found that language skills played a crucial role in integration. More specifically, German adolescents considered language barriers to hinder the interaction between Syrian refugees and Germans. The lack of communication due to limited language skills was regarded as a reason for exclusion.

Furthermore, in young people’s views, morality, social conventions (Beißert and Mulvey, 2022), and group norms (Hitti and Killen, 2015, 2023; Brenick and Romano, 2016) played a crucial role in matters of inclusion and exclusion related to their peers coming from different ethnic backgrounds. Hitti and Killen (2023) conducted three studies with varying intergroup contexts (Arab American/non-Arab American, Asian/non-Asian American, and American/Lebanese) focusing on 12- and 16-year-old adolescents’ judgements of peers who challenged inclusive and exclusive group norms. According to the results, adolescents approved of their peers who challenged exclusive peer norms and supported the inclusion of an ethnic and cultural outgroup, and they disapproved of those who challenged inclusive group norms and supported exclusion. Brenick and Romano (2016) examined the group norms perceived as significant by Jewish-American youth (Mage: 15.70, SD: 1.57) when evaluating the inclusion and exclusion of Arab-American youth across two contexts (peers vs. family at home). The results showed that parental norms were more influential at home, while peer norms were more significant in peer contexts. Also, the role of cultural identity factors was assessed. Adolescents with a strong cultural identity were more likely to consider cultural norms when making decisions about inclusion and exclusion. Finally, focusing on 12 and 16 year old non-Arab Americans’ evaluations of their expectations of inclusivity among Arab American and non-Arab American peer groups toward new peers with similar or different ethnic identities and interests, such as hobbies, Hitti and Killen (2015) found that while stereotypes decreased inclusion, inclusive group norms helped counteract stereotype effects.

In the Swedish school context, focusing on 14- to 15-year-old participants, immigrant background students were found to be rejected more than host population students, and immigrant background students tended to experience more social exclusion in immigrant-sparse classrooms than immigrant-dense classrooms (Plenty and Jonsson, 2017). However, after-school sports programs were found to have a positive impact on social inclusion among ethnic minority students (ages 7–12 and 21–30), as they promoted positive intergroup relations (Carter-Thuillier et al., 2023). In addition, Maor and Gross’s (2023) study among 17- to 21-year-olds, combining ethnic background and religiosity, tested Israeli-born Jewish young people’s attitudes toward minorities (Israeli Arabs and Jews of Ethiopian descent). The researchers found that experiencing social rejection during school years combined with being a member of the dominant group (men and/or European origin vs. women and/or North African/Middle Eastern origin) predicted negative attitudes toward minority ethnic groups in later life.

As a main pattern, these studies highlight how language proficiency and group norms shape youth perceptions of inclusion and exclusions. Language barriers are often seen as key obstacles to integration, while judgments of peers are influenced by moral standards, parental and peer norms, and cultural identity. Inclusive norms can counteract stereotypes, and experiences of social rejection, especially dominant group youth, may predict exclusionary attitudes later in life. These patterns emphasize the need to understand intergroup relations through normative, identity-based, and context-sensitive perspectives.

Our review showed that the theme of social inclusion and exclusion was often studied in relation to other themes. In two articles, social inclusion and exclusion was studied in relation to friendship segregation and forming exclusion ties (Kretschmer and Leszczensky, 2022; Lintner et al., 2023), and in two other studies, social exclusion was coupled with bystander responses (Yüksel et al., 2022; Palmer et al., 2023). Kretschmer and Leszczensky (2022) found religious ingroup bias (ingroup favoritism) among 14- to 15-year-olds being displayed stronger among Muslim girls than Muslim boys, and non-Muslim youth were more open to befriending Muslim girls than Muslim boys. Lintner et al. (2023) focused on 11- to 15-year-old adolescents and examined the integration of Ukrainian refugees in Czech schools and found that Ukrainian students had less friendship ties than Czech classmates and faced neglection rather than rejection. Palmer et al. (2023) found that while British children (ages 8–11) challenged the exclusion of their peers differently on the basis of nationality, more so in the case of national ingroup British and Australian immigrant peers than Turkish, adolescents (ages 13–16) responded to exclusion more equally. Also, Yüksel et al. (2022) found age to play a crucial role, as British participants’ indirect bystander reactions (getting help from others) decreased with ages between 8–10 and 13–15. When witnessing social exclusion, participants were slightly less likely to seek help from a teacher or adult compared to a friend only when the excluder was an ingroup peer (British) but not when the excluder was an outgroup peer (immigrant).

These studies were predominantly conducted from the perspective of host or majority groups, aiming to examine their inclusiveness or exclusiveness towards individuals from diverse ethnic backgrounds, such as refugees. Participants in these studies were typically under 16 years old and school pupils or students. Therefore, findings on social inclusion and exclusion among young people underscore the significance of schools as environments where everyday integration occurs. This may be attributed to the practicality of schools for data collection, but it also emphasizes their role as central locations where young people interact with peers from diverse cultural backgrounds in everyday contexts, shaping their perceptions and experiences of intergroup encounters and individuals from different cultural groups. Consequently, these findings suggest that diversity practices in schools, guided by teachers, are pivotal in promoting feelings, behaviors, and attitudes that support everyday integration. Since schools served as the primary context for examining social inclusion and exclusion, two important perspectives remained underexplored: (1) the role of other meaningful contexts in young people’s lives, such as leisure activities and hobbies, and (2) the experiences of older youth, as the school-based focus led most studies to concentrate on individuals aged 16 or younger.

### 5.2 Cultural or ethnic identity

Nine articles discussed intergroup relations related to integration between young people with immigrant and non-immigrant origins from the perspective of cultural or ethnic identity, including shared or collective identity (Dryden-Peterson, 2010; Banfield and Dovidio, 2013; Bikmen and Sunar, 2013; Jumageldinov, 2014; Çelebi et al., 2014; Brenick and Romano, 2016; Saunders et al., 2022; Carter-Thuillier et al., 2023; Castro et al., 2023). The most utilized theoretical frameworks were intergroup contact theory (Dryden-Peterson, 2010; Bikmen and Sunar, 2013; Brenick and Romano, 2016; Castro et al., 2023) and SIT (Brenick and Romano, 2016). Compared to other commonly studied themes, studies in this thematic category utilized more qualitative (Dryden-Peterson, 2010; Saunders et al., 2022; Castro et al., 2023) and mixed-methods (Jumageldinov, 2014) designs.

The studies focusing on cultural, shared, or collective identity were based on young people’s experiences of integration at school. Therefore, studies on this theme, similar to those on social inclusion and exclusion, emphasized schools as a significant context for everyday integration. In their study on post-secondary school integration experiences among diverse groups of adolescents (ages 18–20) with immigrant origins in Canada, Saunders et al. (2022) found that newcomer ethnic minority students formed a unique cultural identity by integrating their cultural background with experiences in the host country. Two studies (Dryden-Peterson, 2010; Castro et al., 2023) used intergroup dialogue as a method to study cultural and ethnic identities to support integration. Through migration narratives produced in an intergroup dialogue program, Latinx, Black, multi-racial, White, Asian, and Middle Eastern 9th and 10th grade high school students shared and learned from each other’s stories, finding mutual commonalities that enabled building connections between different ethnic groups of young people (Castro et al., 2023). Dryden-Peterson (2010) examined the relationship between a Somali immigrant student and a White U.S. resident student. According to the findings, these high school students built interethnic relationships by transforming casual interactions into meaningful relationships through collective identification built on personal experiences with each other through contact they had in their school. Through dialogical practices, the students challenged and understood each other in new ways, which fostered mutual growth as well as self-understanding in their changing community.

Shared and ethnic identities were also studied in the context of discrimination (Banfield and Dovidio, 2013; Jumageldinov, 2014). In the U.S. context, highlighting a shared American identity, rather than separate ethnic identities, was found to lessen young (Mage: 27.5; SD: 7.4) White people’s awareness of subtle discrimination against their Black peers (Banfield and Dovidio, 2013). In their study conducted in Kazakhstan, Jumageldinov (2014) found that minority (Russians and other ethnic minorities) and majority (Kazakhs) group young people ages 18–31 perceived their relations in different ways. Young people belonging to the majority ethnic group perceived intergroup relations more positively than their minority ethnic group peers, who reported experiences of discrimination and perceived themselves as victims of hidden racism. A solid ethnic identity was found to be a particularly salient buffer against perceived discrimination for ethnic minority young people facing identity threats.

As a general pattern across these studies, young people often construct hybrid identities by integrating elements of their heritage with experiences in the host society. This identity development is supported by dialogical processes, such as intergroup contact and dialogue, which foster mutual understanding and connection across ethnic boundaries. Moreover, findings suggest that strong ethnic identity can help minority youth cope with exclusion and identity threats, aligning with importance of positive ingroup identity emphasized in social identity theory.

As already presented, in two studies, cultural or ethnic identity was studied in relation to social inclusion or exclusion (Brenick and Romano, 2016; Carter-Thuillier et al., 2023). In addition, in two studies, ethnic or shared identity was coupled with outgroup trust (Çelebi et al., 2014) or with attitudes and intergroup contact (Bikmen and Sunar, 2013). The findings of Çelebi et al. (2014) among 18- to 27-year-old participants showed that a stronger ethnic identification tended to lead to lower trust in the outgroup among both Turks and Kurds. In addition, Bikmen and Sunar (2013), focusing on young people ages 18–29, found that a shared religious identity, the quality of stereotypes (negative or positive) toward an ethnic minority group, and the ethnic minority group’s social status formed a basis for the ethnic majority group (Turks) to adjust their interactions with minority groups (Kurds and Armenians). Positive intergroup contact was found to increase the ethnic majority group’s interest in discussing inequality with ethnic minority groups with a shared religious identity (Bikmen and Sunar, 2013).

These findings highlight the dynamic and multifaceted nature of identity in influencing intergroup relations, suggesting that integrated, shared, or collective identities are likely to foster positive everyday integration. Compared to studies in other thematic categories, research on cultural and ethnic identity predominantly involved older participants, typically ages 18–30 years. This trend aligns with the focus of the research, as identity formation is often more pertinent to individuals at the upper end of the youth spectrum (Arnett, 2004). These studies lacked a focus on refugee contexts, as they concentrated on immigrant or ethnic minority youth.

### 5.3 Intergroup contact

The theme of intergroup contact (also conceptualized as social contact or interethnic contact in some studies) was addressed in eight studies (Bikmen and Sunar, 2013; Belet, 2018; Szabó et al., 2020; Wang et al., 2020; Chávez et al., 2021; Hooijsma and Juvonen, 2021; Kaufmann, 2021; Hitti et al., 2023). Intergroup contact theory (Bikmen and Sunar, 2013; Wang et al., 2020; Hooijsma and Juvonen, 2021; Kaufmann, 2021) or the related interethnic contact theory (Belet, 2018) were the most often used theoretical frameworks, and all eight studies utilized quantitative methods.

These studies interpreted contact mainly as a means of reducing intergroup bias in terms of ingroup favoritism drawing from Allport’s (1954) contact hypothesis, and positive contact between different ethnic groups was associated with improved intergroup relations. Contact was mostly defined as person-to-person or group-to-group contact. In addition, Belet (2018) focused both on face-to-face contact and contact in literary intergroup encounters, which were defined as “reading about ingroup members’ encounters with outgroup members” (p. 53). Literary intergroup encounters were described as a beneficial way of reducing intergroup bias in ethnically segregated schools and other settings with limited face-to-face intergroup contact. Students (ages 13–21) who identified themselves as the majority ethnic group (White-Belgian) demonstrated enhanced perceptions of the minority ethnic group (Moroccan Belgian) members when they experienced both in-person and literary interethnic contact. Conversely, the attitudes of students belonging to the minority group were more shaped by literary contact, particularly in classrooms where their interaction with their majority classmates was minimal.

Intergroup contact was also studied with the aim of adding understanding on integration or acculturation (Szabó et al., 2020; Hooijsma and Juvonen, 2021; Kaufmann, 2021). Hooijsma and Juvonen (2021) studied intergroup attitudes between adolescents (Mage: 14.82; SD: 0.56) from different immigrant groups (Dutch-born Turkish, Moroccan, and Surinamese) and societal majority youth (Dutch). According to the findings, outgroup exposure functioned differently across immigrant and Dutch adolescents’ attitudes toward one another. Whereas contact with young Dutch people made the immigrant adolescents’ attitudes toward their Dutch peers more positive, Dutch adolescents’ attitudes toward immigrants differed based on the ethnicity of the immigrant group. Furthermore, Kaufmann (2021) found that contact between 16- to 17-year-old immigrants (in general) and host population Canadian youth increased Canadian adolescents’ support for immigration as well as immigrants’ feelings of belonging in the host country (Canada). Especially intergroup friendships as well as the frequency and friendliness of intergroup contact predicted feelings of belonging (Kaufmann, 2021). Lastly, a study focusing on international students’ (Mage: 23; SD: 4.2) social contacts in Hungary found that students who had mixed social contacts with conational, local, and other international students experienced more psychological wellbeing. In contrast, international students with sparse or mainly international social contacts experienced less psychological wellbeing (Szabó et al., 2020). Therefore, Szabó et al. (2020) concluded that positive contact between immigrant and host youth supported the integration of immigrant youth.

Finally, Wang et al. (2020) studied positive and negative intergroup contact between majority (Han) and minority (Uyghur) group members ages 17–25 in China and their willingness to participate in intergroup interactions with each other (behavioral intentions). The study considered both groups’ perspectives and used social dominance orientation (SDO) as the moderator of contact effects. The findings indicated that positive contact was associated with higher willingness to participate in intergroup interaction among majority group members with a high SDO. Among minority group members, such an association was not found to be moderated by SDO. However, negative contact was associated with lower willingness to participate in intergroup interaction among both high-SDO majority group members and low-SDO minority group members.

As an overarching pattern these studies supported contact hypothesis by showing how positive contact, especially through friendship and positive interactions, was linked to increased feelings of belonging and support for integration. However, the effects of contact varied by group status and context, with minority youth often benefiting more from indirect forms of contact, and negative contact undermining willingness for future intergroup engagement.

Intergroup contact was commonly studied in the context of attitudes and/or friendship (Chávez et al., 2021). Chávez et al. (2021) studied the influence of attitudes (social class prejudice) and perspective-taking abilities (the cognitive component of empathy) on interethnic contact in multicultural Chilean classrooms among seventh graders. According to the findings, adolescents tended to befriend classmates with the same ethnic background. However, those with high perspective-taking abilities (empathy) and low prejudice towards low-social-class peers were more likely to form cross-ethnic friendships. Furthermore, Bikmen and Sunar (2013), focusing on 18- to 29-year-olds, assessed the attitudes of the majority group (Turks) towards two minority groups (Kurds and Armenians) in Turkey, examining the willingness of the majority group to discuss inequality of minority groups. They found that Turks’ attitudes toward the dialogue were based on a shared religious identity, valence of stereotypes (positive/negative), and status of the immigrant group. Finally, Hitti et al. (2023) studied bullying and bystander responses in relation to intergroup contact and found that non-immigrant Americans’ (Mage: 14.54; SD: 0.94) desire for social contact with peers of Arab or Latin immigrant backgrounds significantly influenced the likelihood of intervening in bullying incidents where immigrants were the victims.

Widespread inclusion of intergroup contact in the reviewed articles highlights its continuing prominence as a frequently studied phenomena within the field of intergroup relations. In contrast to studies categorized under social inclusion or exclusion and cultural or ethnic identity, research on intergroup contact more frequently included young participants spanning a broader age range, typically between 13 and 29 years. The majority of these studies focused on intergroup contact between host and immigrant groups, examining perspectives from both groups. This bidirectional approach provides a more comprehensive understanding of the role of intergroup contact in the integration process. However, the context of inter-cultural friendship as a means of enhancing intergroup contact was relatively uncommon in the reviewed studies. Given the significance of peer relationships during youth, and the role of outgroup friendships as one of the key mechanisms for improving intergroup relations (Pettigrew, 1998), this represents an important area for future research.

### 5.4 Attitudes

The fourth largest thematic category was formed by attitudes, which were explored in a total of eight studies (Bikmen and Sunar, 2013; Stark et al., 2015; van Bergen et al., 2017; Bohman and Miklikowska, 2021; Chávez et al., 2021; Hooijsma and Juvonen, 2021; Gönültaş and Mulvey, 2023b; Maor and Gross, 2023). Half of these studies applied intergroup contact theory as the theoretical frame (Bikmen and Sunar, 2013; Stark et al., 2015; Bohman and Miklikowska, 2021; Hooijsma and Juvonen, 2021). Most of these studies utilized quantitative methods (Bikmen and Sunar, 2013; Stark et al., 2015; Bohman and Miklikowska, 2021; Chávez et al., 2021; Hooijsma and Juvonen, 2021; Gönültaş and Mulvey, 2023b; Maor and Gross, 2023), with only one study employing qualitative methods (van Bergen et al., 2017).

The studies provided insights into intergroup attitudes in the following contexts. Bikmen and Sunar (2013) explored the majority ethnic group’s (Turks) attitudes toward two ethnic minority groups (Kurds and Armenians) among 18- to 29-year-old youth and identified three elements contributing to their willingness to enter into a dialogue with minorities. They had more positive attitudes toward discussion with minorities if they (1) shared a religious identity, (2) perceived the minority group positively, and (3) found the minority group belonged to a higher social class category. Studies conducted in educational settings suggested that cross-ethnic friendships and relationships were affected by classroom diversity, which reduced anti-immigrant attitudes and contributed to more positive outgroup attitudes toward immigrants among 13- and 17-year-old Swedish (Bohman and Miklikowska, 2021) and 12- to 13-year-old Dutch (Stark et al., 2015) adolescents and those with high perspective-taking abilities (Chávez et al., 2021). Chávez et al. (2021) found that individual attitudes such as the level of empathy and low prejudice toward low-social-class peers in multicultural classrooms of seventh graders were associated with a higher likelihood for adolescents to form cross-ethnic friendships in Chilean classrooms. Finally, among 16- to 22-year-old youth, van Bergen et al. (2017) studied parent–child (dis)similarity regarding antagonistic (negative and hostile) and egalitarian attitudes toward outgroups among the host population (Dutch) and Muslim immigrant groups (Turkish and Moroccan). While Dutch youth seemed to have similar egalitarian and antagonist attitudes as their parents, attitudes of Muslim immigrants diverged more from their parents’ attitudes. This seemed to be due to different pedagogic relationships, as Muslim youth prone to egalitarian attitudes described that they received sensitive support from their parents when discussing discrimination experiences.

As a main pattern, these studies emphasized the role of social context, especially schools and families, in shaping openness towards ethnic others. In line with intergroup contact theory, positive attitudes are often influenced by perceived similarity, favorable perceptions of minority groups, and social status, which can enhance the quality of contact and reduce bias. Diverse classroom environments and individual traits like empathy and perspective-taking supported meaningful intergroup interactions, such as cross-ethnic friendships, which are key mechanisms for prejudice reduction. Family relations also shaped intergroup attitudes: while majority youth often mirrored their parents’ views, minority youth were more likely to develop independent perspectives, especially when supported in navigating experiences of discrimination—highlighting the importance of supportive contact conditions across different social domains.

Attitudes were predominantly examined in conjunction with intergroup contact (Hooijsma and Juvonen, 2021), threat perception and prejudices (Gönültaş and Mulvey, 2023b), and social exclusion (Maor and Gross, 2023). Hooijsma and Juvonen (2021) found that exposure to outgroups generally enhanced Dutch-born Turkish, Moroccan, and Surinamese adolescents’ attitudes toward Dutch majority youth (Mage: 14.82; SD: 0.56). However, Gönültaş and Mulvey (2023b) indicated that negative media perceptions were directly linked to a greater desire for social distance from Syrian refugees among host group Turkish youth (Mage: 14.81; SD: 0.97 and Mage: 12.19; SD: 1.01). Turkish adolescents with higher negative media perceptions of Syrian refugees felt more threatened, which led to increased prejudice and a stronger desire for social distance. Finally, among majority Israel-born Jewish adolescents (ages 17–21), social rejection was found to be a predictor of negative attitudes toward minorities of Israeli Arabs and Jews of Ethiopian descent among dominant group (men and/or European origin) members (Maor and Gross, 2023).

Once again, the school environment emerged as a central context for everyday integration in studies focusing on attitudes. Classroom diversity, or multicultural classrooms, fostered cross-ethnic friendships and reduced anti-immigrant attitudes. Additionally, the roles of parents and media were identified as potential factors facilitating or hindering integration. Similar to studies on intergroup contact, research on attitudes involved participants across a broad age range, from 12 to 29 years old. These studies also utilized a wide range of population settings, including refugees, immigrants, and ethnic minority groups. The findings highlight the significant role of intergroup contact in shaping attitudes as well as integration processes. Although the reviewed studies employed a variety of contexts, schools remained the dominant setting for data collection. This reliance underscores the need for greater contextual diversity, particularly in everyday environments relevant to young people such as leisure time encountering, when examining attitudes related to integration.

## 6 Discussion

In this scoping review, we examined prior research on young people’s perceptions and experiences of intergroup relations related to (dis)integration, focusing on immigrant and non-immigrant groups. We used intergroup relations as an overarching framework to cover various processes of (dis)integration, such as discrimination, exclusion, inclusion, segregation, and acculturation, explored in previous studies focusing on relations between host/majority and immigrant/minority groups. Through content analysis of 46 studies, we created 17 thematic categories, with the most frequent being social inclusion or exclusion, cultural or ethnic identity, intergroup contact, and attitudes. Most of the studies employed quantitative methods, with intergroup contact theory and social identity theory (SIT) emerging as the dominant theoretical frameworks. In the following, we summarize the main findings, identify the gaps and limitations, propose future research directions, and practical implications.

In summary, this scoping review identified several key factors affecting intergroup relations among ethnically diverse youth. Language proficiency proved crucial, as inadequate skills hindered cross-ethnic interactions and led to exclusion (Beißert et al., 2020). Moral reasoning, social conventions, and group norms significantly influenced the decision-making process related to including or excluding peers from diverse ethnic backgrounds (Beißert and Mulvey, 2022). While stereotypes typically diminished inclusion (Bikmen and Sunar, 2013), they were often counterbalanced by inclusive group norms (Hitti and Killen, 2015).

School settings posed particular challenges, with immigrant students facing greater rejection in classrooms where they were outnumbered (Plenty and Jonsson, 2017; Maor and Gross, 2023). However, after-school sports programs effectively promoted inclusion by fostering intergroup connections (Carter-Thuillier et al., 2023). Cultural identity played a dual role: minority youth developed hybrid identities blending cultural heritage and host-country experiences (Saunders et al., 2022), and intergroup dialogue programs leveraging shared immigrant identities created more inclusive environments (Castro et al., 2023).

Finally, the scoping review highlighted identity’s protective function, showing that strong ethnic identity buffered against discrimination (Jumageldinov, 2014), though it could sometimes reduce outgroup trust (Çelebi et al., 2014). Importantly, shared identity and positive contact facilitated open discussions about inequality and improved majority–minority interactions (Banfield and Dovidio, 2013; Bikmen and Sunar, 2013).

### 6.1 Research gaps and future research

This scoping review disclosed three gaps in previous studies on intergroup relations among young people belonging to different ethnic groups, with a specific focus on integration dynamics: empirical, methodological, and theoretical. Empirically, prior research lacked settings with face-to-face encounters. Although some studies utilized those kinds of settings (e.g., Belet, 2018; Carter-Thuillier et al., 2023; Castro et al., 2023; Dryden-Peterson, 2010; Piipponen, 2023), in most cases, young people were asked about their opinions on or perceptions of different ethnic groups or to contemplate vignettes including different ethnic groups in certain intergroup situations without actual face-to-face encounters. Even though these types of settings add understanding on intergroup phenomena such as attitudes (Bikmen and Sunar, 2013; Stark et al., 2015; van Bergen et al., 2017; Bohman and Miklikowska, 2021; Chávez et al., 2021; Hooijsma and Juvonen, 2021; Gönültaş and Mulvey, 2023b; Maor and Gross, 2023) and prejudice (Chávez et al., 2021; Smith and Minescu, 2021; Gönültaş and Mulvey, 2023b), they may fall short of explaining the actual intergroup behavior taking place in concrete everyday situations where young people interact with peers from different cultural or ethnic backgrounds. After all, conditions are different when thinking about an ethnic group compared to being in actual interaction with them. Future research could employ ethnographic methods or interactive workshops for data collection, as these approaches may better capture real-world contexts and strengthen intergroup relations in integration processes.

In addition, our scoping review identified a notable difference in whose perspective intergroup relations were studied: while 14 studies focused on host country or majority group adolescents’ perspectives on intergroup relations, only 2 examined those of immigrant or minority group adolescents. Although 18 studies included both perspectives and 11 incorporated more diverse groups, our findings highlight the need for greater research attention to immigrant and minority adolescents’ viewpoints.

The aforementioned issue is related to another challenge in the included studies, which is the overlap in defining population contexts in study settings. Majority and minority settings were often used simultaneously with host and immigrant settings. Typically, host groups are majority groups, while immigrant groups tend to be minorities. However, in the context of intergroup relations, this can be confusing. It introduces different levels of analysis, as majority–minority relations often involve more explicitly power dynamics, while host–immigrant relations are more connected to cultural groups as distinct social identity-relevant ingroups and outgroups. While this overlap reflects the inherently interconnected nature of these divisions, it creates challenges for academic purposes by complicating comparisons between qualitatively distinct phenomena.

Another challenge identified in the studies included is the definition of young people. Terms such as adolescent, youth, young people, and sometimes students were used without detailed definitions, leading to vague presentations of age ranges. Only 25 studies provided exact age ranges, while others offered means and standard deviations or simply referred to participants as students. This suggests that many studies were not explicitly focused on youth but rather included young participants without explicitly defining or critically examining the concept of youth itself. This is problematic for two reasons. First, youth is not a homogeneous phase of life or group, and studies should not treat it as such. Detailed definitions of participants are crucial, especially among young people, as age differences can impact intergroup relations, as shown in some studies comparing participants of different ages (e.g., Hitti and Killen, 2015, 2023; Palmer et al., 2023; Yüksel et al., 2022). Second, the lack of detailed age information complicates the interpretation and comparison of results. We consequently urge researchers to provide more precise definitions of their youth study population.

Given the extensive use of schools as data collection environments, it is essential for future research to consider and describe their unique characteristics while collecting data in educational environments. Schools are typically structured with hierarchical relationships between teachers and students, as well as among students themselves. This organizational framework can both facilitate and hinder integration. In schools where teachers implement inclusive practices, these practices may become part of the “hidden curriculum,” promoting values of diversity and inclusivity. Conversely, in schools without such practices, the inherent hierarchy may reinforce prejudices and cultural divisions, leading to imbalanced intergroup relations. Thus, schools can vary significantly as data collection environments, influencing the nature of the data and the results when studying specific phenomena related to intergroup relations, such as prejudice.

On the other hand, overreliance on schools while examining intergroup relations and dynamics on (dis)integration highlights the need to broaden the scope of research contexts. Schools, as formal everyday environments, are shaped by institutional structures that may influence how young people form—or fail to form—intercultural peer relationships. These structures can limit opportunities for genuine encounters between youth from different cultural backgrounds, a gap we also noted in previous research (however, see, e.g., Piipponen, 2023). Therefore, alongside educational settings, future studies should explore other everyday contexts that are locally meaningful to young people, such as hobbies, other leisure activities, and informal gathering spaces. These environments may offer more authentic access to intercultural interactions—or reveal the barriers that prevent them from occurring.

Methodologically, most of the studies (*n* = 34) utilized quantitative methods, only nine used qualitative methods, and one used mixed method. Whereas quantitative studies test theories and assumptions relevant to intergroup relations, qualitative methods enable researchers to gain more in-depth understanding of young people’s thoughts, experiences, and emotions related to intergroup relations as well as meanings constructed to them. Similarly, participatory qualitative methods offer possibilities to implement data collection in actual interethnic encounter settings, enabling the participants to express their perceptions as members of ethnic or cultural minorities and majority groups vis–vis. Such study designs could provide more concrete ways to enhance intergroup relations. This more nuanced understanding of young people’s experiences and perceptions could equip social and societal actors, including educational institutions—with practical tools to facilitate integration.

Another crucial gap in the existing research from a methodological point of view was the scarcity of longitudinal study designs, as most of the studies utilized cross-sectional designs. Only five studies utilized a longitudinal design. Especially when studying intergroup contact, cross-sectional studies leave a central question unanswered: whether the positive effect of intergroup contact found in the study withstands the passage of time in future intergroup contacts. In addition, two key aspects of the contact hypothesis have been noted to benefit from longitudinal designs. The first is the assumed causality from contact to attitude change, which has often been ambiguous due to the prevalent use of cross-sectional designs (Pettigrew, 1998). The second aspect is the generalization from individual to group, that is, understanding what factors enable positive changes in outgroup individuals to extend to the entire outgroup (Brown and Hewstone, 2005). This kind of information provided by longitudinal studies may help social actors to develop and implement more effective measures to facilitate relations between different ethnic groups.

Finally, the third gap we identified in prior studies was theoretical. Theories commonly tested in quantitative studies—such as intergroup contact theory (ICT), social identity theory (SIT), and social reasoning development (SRD)—were prevalent across the reviewed articles, but their application was often left limited to structured, hypothesis-driven approaches. Our review highlights that although aforementioned theories offer valuable insight into mechanisms of intergroup dynamics related to (dis)integration, they may not capture the complexity of young people’s everyday experiences and meaning-making processes in diverse social and cultural contexts. Additionally, theoretical frameworks typically associated with qualitative research were underrepresented. For instance, social representation theory could offer a complementary lens for exploring how young people construct shared everyday understanding of cultural and ethnic groups, intergroup boundaries, and related perceived social positions. Incorporating such approaches could enrich existing frameworks by revealing informal, context-sensitive understanding coming from young people’s voices, lived experiences in everyday contexts of intergroup relations in which dynamics of (dis)integration unfold.

Moreover, future studies could employ more theoretical frameworks at the shared intersection of different theories. For example, the intersection of social identity theory and social representation theory could offer valuable possibilities. While SIT focuses on how individuals define themselves through group memberships, and how these social identities influence intergroup attitudes and behaviors (Tajfel and Turner, 1979), SRT explores how shared understandings—social representations—on social phenomena are constructed within groups (Moscovici et al., 2008), which affects how people make sense of themselves and others. Thus, intersection of these theories would both offer framework combining insights into identity processes and group dynamics, and culture related meaning-making and symbolic boundaries. These kinds of settings would offer more richness in understanding youth experiences on intergroup relations related dynamics on (dis)integration between host and immigrant groups, helping for instance unpack how integration is talked about, imagined and normalized and how these are shaping intergroup behavior.

Our review highlights the need to broaden the empirical, methodological, and theoretical frameworks in future research, in order to include a wider range of approaches for understanding intergroup relations among young people from diverse ethnic backgrounds. The identified research gaps call for the inclusion of actual encounter settings, the adoption of qualitative and mixed-method approaches, and the implementation of theoretical approaches focusing on processes of meaning construction. This would facilitate a more comprehensive understanding of young people’s experiences and perceptions related to intergroup relations. We posit that such an approach would contribute significantly to the comprehension of intricate (dis)integration dynamics and aid in the development of more ethnically and culturally inclusive communities and societies in the future.

### 6.2 Practical implications

Based on the findings of the reviewed studies, we suggest a couple of practical implications for improving intergroup relations between immigrant and non-immigrant youth. Given that schools served as a primary research context across the included studies, the findings offer particularly relevant strategies for educational environments. Classroom diversity or multicultural classrooms provide opportunities for positive intergroup contact, support cross-ethnic friendships, and reduce anti-immigrant attitudes (Bohman and Miklikowska, 2021; Spiel and Strohmeier, 2012). They also facilitate language acquisition for newcomers, enhancing inclusion and integration (Beißert et al., 2020), while intergroup dialogue programs further diminish prejudices and strengthen connections (Castro et al., 2023). These findings support integrative school practices where immigrant students join mainstream classrooms rather than separate ones.

Moreover, after-school programs (e.g., youth centers) should be incorporated alongside formal schooling, as they create vital opportunities for cross-cultural collaboration between students. Initiatives like sports teams, collaborative arts projects, or cultural exchanges in these informal spaces foster organic intergroup interaction, addressing integration gaps that classroom settings alone cannot achieve. Research confirms such programs effectively improve intergroup relations (D’Angelo et al., 2021; Carter-Thuillier et al., 2023), making them essential complements to school-based integration strategies for promoting meaningful, lasting intergroup relations between immigrant and non-immigrant youth.

### 6.3 Limitations

This scoping review had limitations. An intrinsic limitation of scoping reviews is that they detach the findings of the included studies from their relevant contexts. Another limitation of such reviews is that despite a comprehensive data search, a significant amount of literature may remain inaccessible due to a lack of open access or because not all journals are indexed in scientific databases. Additionally, this scoping review lacked the resources to include gray literature or other sources of evidence that could provide valuable insights and broaden the understanding of this specific topic.

Another limitation of the current scoping review is that a significant amount of literature is published in languages other than English. Due to the authors’ language skills, this scoping review only included papers published in English, which may have resulted in findings that overlooked non-English contexts. Furthermore, a notable limitation arose from the lack of clear definitions for key terms such as “native” and “immigrant” within the included studies. This ambiguity is particularly evident concerning individuals with one native parent, as they were inconsistently classified as either native or immigrant. Such inconsistent categorization may significantly affect the interpretation and generalizability of the study findings.

Moreover, it is crucial to recognize the broad spectrum of the term “immigrant,” which encompasses various categories, from voluntary migrants, such as economic and family reunification migrants, to forced migrants, including asylum seekers, refugees, stateless forced migrants, victims of human trafficking, and others. Each category comes with its own distinct definitions and experiences of immigration, thereby adding complexity to the interpretation of the results.

## Data Availability

The original contributions presented in the study are included in the article/Supplementary material, further inquiries can be directed to the corresponding author.

## Footnotes

1 Covidence systematic review software, Veritas Health Innovation, Melbourne, Australia. Available at www.covidence.org.

2 We also included 12 articles that had some participants younger than 13. In six of these cases, age ranges were 10–17 (Beißert et al., 2020), allegedly 12–13 (Chávez et al., 2021), 11–15 (Lintner et al., 2023; Mazzone et al., 2018), 10–14 (Smith and Minescu, 2021), and 12–13 (Stark et al., 2015), and in six other cases, studies included data with two different age ranges: 7–12 and 21–30 (Carter-Thuillier et al., 2023), 12 and 16 (Hitti and Killen, 2015, 2023), 8–11 and 13–16 (Palmer et al., 2023), 10–11 and 14–19 (Spiel and Strohmeier, 2012), and 8–10 and 13–15 (Yüksel et al., 2022). In addition, we included one article that had some older participants within the age range 18–31 (Jumageldinov, 2014). We included these studies as, according to our interpretation, they essentially met our age range criteria, despite the fact that some participants were slightly younger or older.

3 The book chapter by Spiel and Strohmeier (2012) was not included in the quality appraisal process.
