# Supplementary material for: Intergroup relations and dynamics of (dis)integration between youth with immigrant and non-immigrant origins: a scoping review
Source: Front Psychol. 2025 Nov 26;16:1681385. doi: 10.3389/fpsyg.2025.1681385 (PMC12689515; doi:10.3389/fpsyg.2025.1681385)
Supplement: Supplementary file 1 [file Table_1.DOCX]

Table 1 presents the data extracted from the included studies

| **Author, Year, Country** | **Aims of the study** | **Main focus on intergroup relations** | **Population context**  1. Host population and immigrants, refugees or other ethnic group  2. Majority and minority group  3. Diverse population context | **Theoretical framework** | **Study Design, and number of participants** | **Main findings** |
| --- | --- | --- | --- | --- | --- | --- |
| Aral et al., (2022), Germany | To examine the link between cultural diversity approaches and adolescents' prosocial intentions and behavior towards refugee youth. | Prosocial intentions/behavior | 1. From host group German’s (of these 31% were of non-immigrant descent, 3% first generation immigrants, 69% second and third generation immigrants but all sample have had a German citizenship) perspective towards refugees on cultural diversity approaches | Cultural diversity approaches | Quantitative, 503 | Greater heritage and intercultural learning were linked to positive attitudes and prosocial intentions towards refugee youth. Similarity predicted prosocial intentions and willingness to donate. Identity inclusiveness didn't mediate the relationship between cultural diversity approaches and prosocial intentions. |
| Banfield and Dovidi, (2013), United States | To study how representations of group identities impact White individuals' recognition of discrimination against Black individuals and their willingness to protest on behalf of them. | Cultural or ethnic identity | 2. From majority group White residents’ perspective on perceptions of discrimination against minority Black Americans | Prior literature | Quantitative; Experimental, 118 | Emphasizing common American identity reduces Whites' recognition of subtle discrimination against Black Americans. External threats to this identity can moderate Whites' sensitivity to injustices against Blacks, potentially increasing recognition of racial bias. |
| Beißert et al., (2020), Germany | To study adolescents' decisions on social inclusion based on peers' refugee status and language skills, aiming to understand refugee youth's social integration. | Social inclusion or exclusion | 1. From host group German’s perspective on decisions on social inclusion and exclusion of Syrian refugees | Social Reasoning Development (SRD) | Quantitative; Experimental, 100 | Adolescents were less inclusive towards refugees with poor German skills. Language proficiency was crucial for integration, with females generally more inclusive. Positive attitudes towards foreigners justified inclusion. Language barriers hindered interaction, with less attribution of exclusion to refugees with poor language skills. |
| Beißert and Mulvey, (2022), Germany | To examine adolescents' decisions and reasoning about including Syrian peers in Germany, comparing their decisions with group expectations and norms. | Social inclusion or exclusion | 1. From host group German’s perspective on decisions and reasonings on including Syrian peers | Social Reasoning Development (SRD) | Quantitative, 100 | Adolescents thought they were more inclusive of Syrian peers than German peers, citing moral and social conventions. They expected their group to favor German peers over Syrian peers, based on group dynamics and psychological factors. |
| Belet, (2018) Belgium | To explore how literary interethnic contact influences high school students' attitudes toward ethnic minorities and awareness of discrimination. | Intergroup contact | 1. From the perspective of both host group white-Belgian and immigrant group Moroccan-Belgian on perspectives on interethnic contacts, prejudice, and discrimination | Interethnic contact theory (Relates to Intergroup Contact Theory) | Quantitative; Experimental, 977 | Literary intergroup contact effectiveness varies by face-to-face interaction quality and students' ethnic self-identifications. Belgian students improved attitudes toward Moroccans with both types, while non-Belgians were more influenced by literary contact in less diverse classrooms. |
| Bikmen and Sunar, (2013), Turkey | To explore majority group Turkish attitudes towards dialogue with minority Kurds and Armenians, examining willingness to discuss power inequalities among minority groups and its predictors. | Attitudes | 2. From the perspective on majority group Turks on willingness to discuss inequality with minority groups Kurds and Armenians | Intergroup Contact Theory | Quantitative, 164 | Participants equally discussed commonality but less engaged in power talk, especially with Armenians. Religious identification moderated, particularly with Kurds. Positive contact boosted interest in power talk. Majority group members adjusted interactions based on stereotypes, status, and shared identity. |
| Bohman and Miklikowska, (2020), Sweden | Examining the long-term effects of classroom diversity on anti-immigrant attitudes and cross-ethnic friendships in adolescence, including direct and indirect effects and their longevity. | Attitudes | 2. From the perspective of majority group Swedish on anti-immigrant attitudes towards immigrant minorities in general | Intergroup contact theory | Quantitative, 661 | Classroom diversity has limited direct effects on anti-immigrant attitudes. It increases cross-ethnic friendships, linked to lower anti-immigrant attitudes. This effect persists even after adolescents change schools. |
| Brenick et al. (2016), United States | To study how peer and parent intergroup norms about cultural out-groups relate to evaluations of intergroup exclusion, especially regarding a youth's cultural identity. | Social inclusion or exclusion | 3. From minority group Jewish American’s points of view towards another minority group Arab-American on how perceived peer and parent out group norms relate to intergroup exclusion and inclusion | Intergroup Contact Theory (Social Identity Theory) | Quantitative, 241 | Perceived group norms shape adolescents' intergroup decisions. Extended contact, like cross-group friendships, positively influences in-group norms and behaviors. Addressing group-specific norms is crucial for effective interventions promoting inclusivity. |
| Brenick et al., (2012), Germany | To examine factors influencing perceptions of discrimination in ethnic German migrant youths from the former Soviet Union. | Discrimination | 1. From the perspective of both host group German and ethnic German migrants (from former Soviet Union) on perceptions on discrimination | Cultural Identity Theory | Quantitative, 1457 | Aussiedler adolescents reported more discrimination in schools with higher Aussiedler percentages and negative attitudes. Ingroup orientation and perceived discrimination were stronger in schools with fewer Aussiedler students. |
| Carter-Thuillier et al., (2023), Spain; Chile | To explore how school sports programs promote social inclusion and intercultural understanding among immigrant and native students, fostering positive relations and cultural identity in schools. | Social inclusion or exclusion | 1. From the perspective of both host groups (study 1. Spanish, Study 2. Chilean) and immigrants (from several countries) on social inclusion and intercultural understanding | Intercultural competence | Qualitative, 169 | SCSP programs enhance social inclusion among minority students through empathy and collaboration, fostering positive intercultural relations and new social networks. Teachers need specific training to manage intercultural conflicts effectively. |
| Castro et al., (2023), United States | To explore how an intergroup dialogue program fosters connection across immigrant backgrounds in a high school, focusing on a shared identity of migration. | Cultural or ethnic identity | 3. The study involved non-immigrant Americans and immigrant background youths (Latinx, Black, Multiracial, White, Asian, and Middle Eastern, and first and second generation immigrant students) and explored intergroup connection and identity from everyone’s perspective. | Intergroup contact theory | Qualitative, 159 | The program enabled self-disclosure and learning from migration narratives, fostering connection among students. Connection varied, with the program being most effective for majority members, especially second-generation immigrant-origin and Latinx youth. |
| Çelebi et al., (2014), Turkey | To study how conflict perceptions influence out-group trust, considering ethnic and national identification. | Out-group trust | 1. From the perspective of both host group Turks and ethnic minority group Kurds on out group trust | No specific theory (focused on concepts of out-group trust and conflict) | Quantitative, 625 | Out-group trust was low, with Turks trusting Kurds less. Both blamed each other, hindering reconciliation. Stronger ethnic identification led to lower trust for both, while higher national identification increased trust for Kurds but decreased it for Turks. |
| Chávez et al., (2021), Chile | To examine how attitudes like perspective-taking and social class prejudice influence interethnic contact in Chilean classrooms with many immigrant students. | Attitudes | 1. From the perspective of both host group Chilean and non-Chilean from several South American countries on attitudes | Social Cognitive Theory | Quantitative, 242 | Adolescents reciprocate friendships and prefer classmates of the same ethnicity. Perspective-taking influences cross-ethnic friendships. Prejudice towards low social class peers has little impact. |
| Dryden-Peterson, (2010), United States | To examine one relationship between a Somali immigrant and a White long-time resident using portraiture methodology to reflect theoretical issues. | Cultural or ethnic identity | 1. From the perspective of both host group’s White long time US resident and Somali immigrant on relationship and cultural identity | Social capital theory, Equal status contact theory (Relates to Intergroup Contact Theory) | Qualitative, 2 | Participants in the new immigrant destination school formed bridging relationships based on collective identification, fostering mutual challenge and growth through dialogue, particularly about race, to understand themselves and their evolving community. |
| Gönültaş and Mulvey, (2023a), Turkey | To study bystander responses to bias-based bullying in Turkey during the refugee crisis, comparing intergroup and intragroup bullying scenarios. | Bystander responses | 1. From the perspective of host group Turkish on bystander responses towards Syrian refugees | Social Reasoning Developmental (SRD) | Quantitative, 587 | Adolescents oppose bullying but tolerate intergroup bias-based bullying more than intragroup bullying. Social-cognitive factors like empathy and Theory of Mind influence responses, while intergroup factors like prejudice and desired social distance from Syrian refugees affect bystander reactions. |
| Gönültaş and Mulvey, (2022), Turkey | This study examines how ingroup and outgroup ToM relates to bystanders' judgments of intergroup bullying of refugee peers, offering insights into when youths challenge and stop such bullying. | Bystander responses | 1. From the perspective of host group Turkish on bystander judgements of intergroup bullying of Syrian refugee peers | Social Cognitive Theory (Intergroup Contact Theory) | Quantitative, 587 | Participants with stronger Theory of Mind (ToM) skills understood bullying reasons better. Those with robust outgroup ToM grasped intergroup bullying reasons like discrimination and prejudice. |
| Gönültaş and Mulvey, (2023b), Turkey | To explore how negative media perception influences Turkish adolescents' discriminatory attitudes towards Syrian refugees in Turkey. | Threat (identity, perception etc.) | 1. From the perspective of host group Turkish on threat perception and prejudice towards Syrian refugees | Prior literature | Quantitative, 587 | Negative media perceptions were linked to a desire for greater social distance from Syrian refugees, driven by increased threat perception and resulting prejudice. The study underscores media's role in shaping intergroup attitudes. |
| Hitti and killen, (2015), United States | To study age-related differences in how non-Arab American children prioritize group membership versus individual characteristics in selecting peer group members. | Social inclusion or exclusion | 3. Non-Arab Americans assessed their expectations about the inclusivity of Arab American and non-Arab American peer groups toward new peers | Social domain theory | Quantitative, 296 | Non-Arab American children expected out-groups to prioritize ethnicity over shared interests, valuing shared interests more in their own groups. Stereotypes influenced inclusion, but inclusive group norms countered this. Older participants were less inclusive and more likely to stereotype. |
| Hitti and killen, (2023), United States | To investigate how adolescents from different ethnic backgrounds evaluate deviant behaviors and whether ingroup bias influences these evaluations. | Social inclusion or exclusion | 3. This study on exclusive and inclusive group norms included 3 different studies:  1. non-Arab American participants responding to an Arab American/non-Arab American intergroup context.  2. non-Asian and Asian  American participants responding to an Asian/non-Asian American intergroup context. 3. Lebanese participants responding to an American/Lebanese intergroup context. | Social Reasoning Development (SRD), Intergroup contact model (Relates to Intergroup Contact Theory) | Quantitative, 597 | Non-Asian and Asian American adolescents approve inclusive deviants challenging exclusive norms but disapprove of the reverse. Non-Asian Americans show ingroup bias, while Lebanese Arab youth consistently approve inclusive deviants. |
| Hitti et al., (2023), United States | To understand bystander responses to bullying of immigrant-origin victims and investigate the role of contact with immigrants. | Bullying | 3. From the perspective of immigrant and non-immigrant American students towards Arab or Latin immigrants on bystander responses to bullying | Social Reasoning Development (SRD) | Quantitative, 168 | Desire for social contact with immigrant peers strongly influences bystander intervention in bullying involving immigrant victims. |
| Hooijsma Juvonen, (2021), Netherlands | To examine intergroup attitudes among immigrant and majority youth, exploring factors like exposure to out-group classmates and formation of out-group friendships. | Intergroup contact | 1. From the perspective of both host group Dutch and Dutch-born Turkish, Moroccan, and Surinamese immigrant youth on intergroup contact and formation of out-group friendships | Intergroup Contact theory, Social identity theory | Quantitative, 2680 | Results varied among immigrant and majority youth across cultural groups. Out-group exposure generally improved immigrant adolescents' attitudes toward the majority. |
| Jumageldinov, (2014), Kazakhstan | To address the role of ethnic identification among the Kazakh majority and minorities in post-USSR Kazakhstan, focusing on perceived discrimination. | Cultural or ethnic identity | 1. From the perspective of both host group Kazakh and immigrants on ethnic identification | Social identity theory | Mixed method, 371+18 | Russian and other minorities in Kazakhstan perceive discrimination differently from the majority, partly due to their asymmetric relationship. Ethnic identity is particularly important for minorities facing identity threats. |
| Kaufmann, (2021), Canada | To capture youths' intergroup contact and perspectives on immigration, analyzing their influence on integration rates and support for immigration. | Intergroup contact | 1. From the perspective of both host group Canadians and immigrants on intergroup relations and support for migration | Intergroup contact theory | Quantitative, 222 | Results support contact theory and the multiculturalism hypothesis. Contact and friendships between immigrant and Canadian-born youth correlate with belonging and support for immigration, while perceptions of diversity as threatening predict lower immigration support. |
| Kisfalusi et al., (2020), Hungary | To investigate associations between bullying and ethnicity among non-Roma majority and Roma minority Hungarian students. | Bullying | 2. From the perspective of both majority Non-Roma’s and Roma minority on bullying | Social identity theory (misfit theory) | Quantitative, 347 | Bullying and victimization in majority vs. minority setting. Both self-declared Roma and non-Roma students more likely to bully perceived Roma peers than non-Roma peers. Victim's self-declared ethnicity not significantly linked to bullying likelihood. |
| Korem and Horenczyk, (2015), Israel | To examine social strategies in intercultural relations among young immigrants with threatened identities, focusing on their coping patterns with non-immigrant group members. | Threat (identity, perception etc.) | 1. From immigrants (Ethiopian) perspective towards non-immigrants on coping patterns of immigrants with threatened identities | Prior literature | Qualitative, 12 | Immigrants often adopt host culture perspectives on social norms, exhibiting complex social behaviors. This spectrum suggests dynamic trial-and-error processes, reflecting unique intercultural competence complexities. |
| Kretschmer and Leszczensky , (2022), Germany | The study examined whether religious friendship segregation arises because of Muslims’ in-group bias or because of non-Muslims’ reluctance to befriend them. | Friendships | 2. From the perspective of both majority non-Muslims Germans’ and minority Muslims’ on religious friendship segregation | Prior literature | Quantitative, 3194 | Muslim girls show strong in-group bias, but non-Muslim youth are open to befriending them. Conversely, Muslim boys display weaker in-group bias, yet non-Muslim youth are less inclined to befriend them than non-Muslims. |
| Lintner et al., (2023), Czech Republic | To examine the integration of Ukrainian refugees in Czech school networks post-Russian invasion, focusing on friendship and exclusion ties and the impact of classroom ethnic composition. | Friendships | 1. From the perspective of both host group Czech and Ukrainian refugees on friendship formation | Social integration, Network theory | Quantitative, 266 | Social networks formed along ethnic lines with strong homophily. Ukrainian students tended to have fewer friendship ties than Czech classmates and were neglected rather than explicitly rejected. |
| Maor and Gross, (2023), Israel | To investigate if social rejection influences negative attitudes towards minority groups in adulthood based on group dominance and religiosity levels. | Social inclusion or exclusion | 2. From majority Israel-born Jewish perspective on minorities Israeli Arabs and Jews of Ethiopian descent on attitudes towards minorities | Social Dominance Theory, Social Identity Theory | Quantitative, 300 | Social rejection predicted negative attitudes toward minority groups among dominant group members and highly religious individuals. It influenced acceptance or rejection of minority groups based on school environment and religious beliefs. |
| Mazzone et al., (2018), Italy | To explore students' perspectives on bullying towards same-country and immigrant peers, including their views on the motives for bullying. | Bullying | 1. From the perspective of both host group Italian and immigrants (from different contents) on bullying | Socio-ecological model of human development (Brofenbrenner) | Qualitative, 35 | Socializing deviance underlies both forms of bullying, reflecting shared peer group beliefs about the victim's deviant traits. Sub-categories include rejecting newcomer, physical, and personality deviance. |
| Munayand Horenczyk, (2014), Israel | To examine changes in acculturation orientations and cultural distances in 1998 and 2009 among Palestinian Christian Arab adolescents in Israel. | Acculturation | 2. From the perspective of minority Palestinian Christian Arabs on Muslim Arabs and Israeli Jews as majorities on minority’s acculturation orientation to majorities | Acculturation orientation model (Berry (1997)) | Quantitative, 237 | Separation was the strongest orientation towards both majority groups and the Integration attitude was weakening. Palestinian Christian Arabs perceive Israeli Jewish culture as less Western, increasingly identify with their own culture. |
| Palmer et al., (2023), Britain | To study age-related differences in bystander reactions to peer exclusion among British and immigrant peers. | Bystander responses | 1. From host group British perspective (self-reported British identity) on differences in bystander reactions in the context of peer exclusion of national ingroup  (British) and immigrant outgroup (Australian or Turkish) peers | Social Reasoning Developmental (SRD), Social exclusion model | Quantitative, 303 | Children were more likely to challenge exclusion for British or Australian peers than for Turkish peers. Adolescents responded equally regardless of peer nationality. |
| Piipponen, (2023), Belgium | To explore meaningful intercultural encounters and perceived learning from intercultural story exchange. | Intercultural encountering | 3. Study focused on encountering experiences among international (from 14 nationalities) in international school in Belgium without any specific “counter” setting, such as host/immigrants or majority/minority. | Deweys experiential learning, Theories of interculturality | Qualitative, 18 | The study found that intercultural story and drawing exchanges in primary school are meaningful for children. Participants perceived personal and communal growth and developed friendships with distant partners. |
| Plenty and Jonsson, (2017), Sweden | To study how immigrant status and classroom immigrant density influence social exclusion outcomes: victimization, isolation, and rejection. | Social inclusion or exclusion | 1. From the perspective of both host group Swedish and immigrants (Second generation European background immigrants, second generation non-European, first generation European and first generation non-European immigrants) on how classroom immigrant density influence social exclusion | Social identity theory (misfit theory) | Quantitative, 4795 | Immigrant students faced more rejection than majority youth, with first-generation non-European immigrants most isolated. Social exclusion was higher in immigrant-sparse classrooms, while victimization increased with immigrant density for majority youth. |
| Priest et al., (2014), Australia | To systematically review 30 years of research on ethnic-racial socialization processes for minority and majority children and young people. | Ethnic-racial socialization | systematic review | Socio-ecological approach (Bronfenbrenner) | Review | Most studies focus on African American children's ethnic-racial socialization by parents, but emerging research on diverse groups stresses considering various factors and agents shaping messages to children. |
| R'boul, et al., (2023), Morocco | To explore majority-group acculturation in Morocco within South-South acculturation and subalternity mobilization. | Acculturation | 1. From host group Moroccan’s perspective on Sub-Saharan immigrants (Burundi, Ghana, Guinea Bissau, Niger, Nigeria, Senegal, and Sudan) on acculturation | Critical approach to acculturation | Qualitative, 35 | Moroccan students express support for Sub-Saharan presence on campuses, but not necessarily willing to adopt their perspectives. Majority group acculturation requires deeper understanding of migrants' values beyond local hospitality. |
| Rientiesand Nolan, (2014), United Kingdom | To explore how international students build and maintain relations with co-national, multi-national, and/or host-national students over time. | Social networks | 3. From international student’s perspective: how they built/maintain relations with co-nationals, multi-nationals and UK host national students | Social Network Theory, Social Capital theory | Quantitative, 592 | Students formed networks based on shared backgrounds, with limited integration. Group activities briefly boosted cross-cultural interaction. Co-national groups provided belonging but might hinder adjustment. Other students acted as bridges. |
| Saunders, et al., (2022), Canada | An arts-based engagement ethnography explored post-secondary integration experiences of 10 emerging adults from a person-first perspective. | Cultural or ethnic identity | 3. Immigrants’ integration experiences in Canada without any specific “counter” setting, such as host/immigrants or majority/minority. | Art-Based Ethnographic Approach, Social Justice Framework | Qualitative, 10 | Post-secondary newcomers integrate origin culture with the Canadian experience, forming a unique identity. Acculturation, biculturalism, and managing familial expectations are key. Navigating the newcomer experience in the classroom is crucial. |
| Smith, and Minescu, (2021), Ireland | To explore how perceived norms from different social groups influence children's anti-refugee bias and the mechanisms involved. | Prejudice | 3. Native Irish’s and non-Irish’s prejudices towards refugees without any specific “counter” setting, such as host/immigrants or majority/minority. | Social identity theory, Intergroup contact theory | Quantitative, 266 | Children's anti-refugee bias was significantly influenced by family and religious group norms, shaping their perceptions and interactions with refugees. Peer norms had no significant impact, suggesting stronger influence from family and religious communities than peers. |
| Spiegler et al., (2024), England; the Netherlands; Germany; Sweden | To study how classroom ethnic diversity relates to peer victimization among European students, considering the role of teacher support. | Cultural or ethnic diversity | 2. From the perspective of both majority students (student and both parents born in host country) and ethnic minority students (student and/or at least one parents born abroad) on how classroom ethnic diversity relates to peer victimization. | Balance of power, Ethnic competition theory | Quantitative, 18716 | Diversity correlated with reduced victimization among ethnic minority students. Teacher support was linked to less victimization for both ethnic minority and majority students, especially in classrooms with varying levels of diversity. |
| Spiel and Strohmeier, (2012) (Not applicable) | The chapter reviews peer relations across race, ethnicity, and culture, covering friendships and bully-victim behavior. | Friendships | 1. Book chapter. Focusing on peer relations in general including also relations between immigrant and native youth in different countries. | Not applicable | Book chapter | Positive peer relations are vital for immigrant and non-immigrant youth in multicultural schools. Interventions targeting all students can promote them effectively. Comprehensive research should study immigrant groups across host countries and in their country of origin, incorporating contextual factors and longitudinal analyses. |
| Stark et al., (2015), Netherlands | To explore how relationships between students from different ethnic groups in mixed schools influence outgroup attitudes. | Attitudes | 2. From majority groups Dutch’s perspective on ethnic minority groups (from 65 different ethnic groups) on interethnic attitudes. | Intergroup contact theory | Quantitative, 728 | The quantity and quality of relationships with outgroup classmates significantly affect outgroup attitudes. More liked classmates lead to positive attitudes, while more disliked classmates lead to negative attitudes. |
| Szabó et al., (2020), Hungary | To explore international students' social interactions with co-nationals, locals, and other internationals at and outside of school. | Intergroup contact | 3. From international students’ perspective. Social contact configurations in Hungary with co-nationals, locals and other internationals. | Acculturation theory by Berry (1997), Person-Centered Social Contact Approach | Quantitative, 291 | Five contact profiles showed varying engagement with co-nationals, internationals, and local Hungarians, associated with acculturation strategies like separation, marginalization, integration, and assimilation. Students with mixed social contact had positive psychological outcomes, while isolated or predominantly international contacts had poorer adaptation. |
| vanBergen et al., (2017), Netherlands | To explore the attitudes of Muslim minority and native youth and their parents, focusing on antagonistic versus egalitarian perspectives. | Attitudes | 1. From the perspective of both host group Dutch and Muslim immigrants on intergroup attitudes and perceived parental socialization. | Social cognitive learning theory (Bandura, 1986), Ethnic socialization strategies Hughes et al. (2008) | Qualitative, 22 | Youth held varied attitudes towards out-groups. Minority youth diverged more from parental attitudes than majority youth. Egalitarian minority youth received sensitive parental responses, while antagonistic ones lacked guidance, particularly regarding discrimination and Islamophobia. |
| Wang et al., (2020), China | To investigate if positive and negative intergroup contact associations with behavioral intentions are moderated by social dominance orientation (SDO). | Intergroup contact | 2. From both majority Han’s and minority Uyghur’s perspective on intergroup contact. | Intergroup Contact Theory, Social Dominance Theory | Quantitative, 325 | Positive contact between majority and minority groups fosters positive intentions among majority members with high-SDO. Negative contact was associated with lower willingness to intergroup interactions among high-SDO majority and low-SDO minority group members. |
| Yüksel et al., (2022), Britain | To explore developmental changes in British children's reactions to social exclusion and their reasoning, considering group memberships involved. | Bystander responses | 1. From host group British’s perspective towards British and immigrants on indirect bystander reactions when witnessing social exclusion of in-group or out-group peer. | Social Reasoning Developmental (SRD) | Quantitative; Experimental, 424 | Participants' indirect bystander reactions decreased with age. Children trusted teachers and friends, while adolescents cited group loyalty and psychological factors. |
| Zhou et al., (2022), United States | To explore if cross-racial friendships affect the link between discrimination and depression among Korean domestic and international students. | Friendships | 3. Cross-race friendships were studied with majority and minority peers among Korean domestic and international students in the United States. | Intergroup contact theory | Quantitative, 219 | Cross-race friendships with majority peers increase school belonging and reduce discrimination's negative impact. They don't significantly affect depressive symptoms. Cross-race friendships with minority peers show no significant effects. |
