# Supplementary material for: Intergroup relations and dynamics of (dis)integration between youth with immigrant and non-immigrant origins: a scoping review
Source: Front Psychol. 2025 Nov 26;16:1681385. doi: 10.3389/fpsyg.2025.1681385 (PMC12689515; doi:10.3389/fpsyg.2025.1681385)
Supplement: Supplementary file 2 [file Table_2.DOCX]

Table 2: JBI Critical Appraisal Checklist for Qualitative Research

| Author, Year, Country | Title | Is there congruity between the stated philosophical perspectives and the research methodology? | Is there congruity between the research methodology and the research question or objectives? | Is there congruity between the research methodology and the methods used to collect data? | Is there congruity between the research methodology and the representation and analysis of data? | Is there congruity between the research methodology and the interpretation of results? | Is there a statement locating the researcher culturally or theoretically (positionality of the researcher)? | Is the influence of the researcher on the research, and vice-versa, addressed? | Are participants, and their voices adequately represented? | Is the research ethical according to current criteria or, for recent studies, and is there evidence of ethical approval by an appropriate body? | Do the conclusions drawn in the research report flow from the analysis, or interpretation, of the data? | Were the conclusions valid given the data represented? |
| --- | --- | --- | --- | --- | --- | --- | --- | --- | --- | --- | --- | --- |
| Carter-Thuillier et al., (2023), Spain; Chile | After-school sports programmes and social inclusion processes in culturally diverse contexts: Results of an international multicase study | Yes | Yes | Yes | Yes | Yes | Unclear | Unclear | Yes | Yes | Yes | Yes |
| Castro et al., (2023), United States | "Everyone has their story": Intergroup dialogue's potential to cultivate connection through the sharing of migration narratives | Yes | Yes | Yes | Yes | Yes | Yes | Yes | No | Yes | Yes | Yes |
| Dryden-Peterson, (2010), United States | Bridging home: Building relationships between immigrant and long-time resident youth | Yes | Yes | Yes | Yes | Yes | Yes | Yes | Yes | No | Yes | Yes |
| Jumageldinov, (2014), Kazakhstan | Ethnic Identification, Social Discrimination and Interethnic Relations in Kazakhstan | Unclear | Yes | Yes | Yes | Yes | No | No | Unclear | Unclear | Yes | Yes |
| Korem and Horenczyk, (2015), Israel | Perceptions of social strategies in intercultural relations: The case of Ethiopian immigrants in Israel | Unclear | Yes | Yes | Yes | Yes | Yes | Unclear | Yes | Yes | Yes | Yes |
| Mazzone et al., (2018), Italy | "Judging by the cover": A grounded theory study of bullying towards same country and immigrant peers | Yes | Yes | Yes | Yes | Yes | No | No | Yes | Yes | Yes | Yes |
| Piipponen, (2023), Belgium | Studentsâ€™ perceptions of meaningful intercultural encounters and long-term learning from a school story exchange | Yes | Yes | Yes | Yes | Yes | Unclear | Yes | Yes | Yes | Yes | Yes |
| R'boul, et al., (2023), Morocco | South-South acculturation: Majority-group studentsâ€™ relation to Sub-Saharan students in Moroccan universities | Yes | Yes | Yes | Yes | Yes | Yes | Yes | Yes | Unclear | Yes | Yes |
| Saunders, et al., (2022), Canada | Bridging Cultural Identities: Examining Newcomerâ€™s Post-Secondary School Integration Experiences Through an Arts-Based Ethnographic Approach | Yes | Yes | Yes | Yes | Yes | Yes | Yes | Yes | Yes | Yes | Yes |
| vanBergen et al., (2017), Netherlands | "Us Against Them" or "All Humans Are Equal": Intergroup Attitudes and Perceived Parental Socialization of Muslim Immigrant and Native Dutch Youth | Yes | Yes | Yes | Yes | Yes | No | Yes | Yes | Unclear | Yes | Yes |
