# Supplementary material for: Intergroup relations and dynamics of (dis)integration between youth with immigrant and non-immigrant origins: a scoping review
Source: Front Psychol. 2025 Nov 26;16:1681385. doi: 10.3389/fpsyg.2025.1681385 (PMC12689515; doi:10.3389/fpsyg.2025.1681385)
Supplement: Supplementary file 3 [file Table_3.DOCX]

Table 3: JBI Critical Appraisal Checklist for Systematic Reviews and Research Synthesis

| Author, Year, Country | Title | Is the review question clearly and explicitly stated? | Were the inclusion criteria appropriate for the review question? | Was the search strategy appropriate? | Were the sources and resources used to search for studies adequate? | Were the criteria for assessing (quality) studies appropriate? | Was critical appraisal conducted by two or more reviewers independently? | Were there methods to minimize errors in data extraction? | Were the methods used to combine studies appropriate? | Was the likelihood of publication bias assessed? | Were recommendations for policy and/or practice supported by the reported data? | Were the specific directives for new research appropriate? |
| --- | --- | --- | --- | --- | --- | --- | --- | --- | --- | --- | --- | --- |
| Priest et al., (2014), Australia | Understanding the complexities of ethnic-racial socialization processes for both minority and majority groups: A 30-year systematic review | Yes | Yes | Yes | Yes | Yes | Yes | Yes | Yes | Yes | Yes | Yes |
| Spiel and Strohmeier, (2012) (Not applicable) | Peer relations in multicultural schools | Yes | Yes | Not applicable | Yes | Not applicable | Not applicable | Not applicable | Not applicable | Not applicable | Yes | Yes |
