# Supplementary material for: Intergroup relations and dynamics of (dis)integration between youth with immigrant and non-immigrant origins: a scoping review
Source: Front Psychol. 2025 Nov 26;16:1681385. doi: 10.3389/fpsyg.2025.1681385 (PMC12689515; doi:10.3389/fpsyg.2025.1681385)
Supplement: Supplementary file 4 [file Table_4.DOCX]

Table 4: JBI Critical Appraisal Checklist for Quantitative Research

| Author, Year, Country | Title | Was the sample frame appropriate to address the target population (was the sample representative of the population)? | Were study participants sampled in an appropriate way (bias related to selection and sampling)? | Was the sample size adequate? | Were the study subjects and the setting described in detail (comprehensiveness of reporting)? | Were valid methods used for the identification of the condition (Bias related to assessment, detection, and measurement of the outcome)? | Was the condition measured in a standard, reliable way for all participants (Bias related to assessment, detection, and measurement of the outcome)? | Was there appropriate statistical analysis (Statistical conclusion validity)? | Was the response rate adequate and, if not, was the low response rate managed appropriately (Bias related to selection and allocation)? |
| --- | --- | --- | --- | --- | --- | --- | --- | --- | --- |
| Aral et al., (2022), Germany | Cultural diversity approaches in schools and adolescents' willingness to support refugee youth | Yes | Yes | Yes | Yes | Yes | Yes | Yes | Yes |
| Banfield and Dovidio, (2013), United States | Whites' perceptions of discrimination against Blacks: The influence of common identity | No | No | Unclear | Yes | Yes | Yes | Yes | Unclear |
| Beissert et al., (2020), Germany | Social Inclusion of Refugee and Native Peers Among Adolescents: It is the Language that Matters! | Yes | No | Yes | Yes | Yes | Yes | Yes | Unclear |
| Beissert et al., (2022), Germany | Inclusion of Refugee Peers – Differences Between Own Preferences and Expectations of the Peer Group | Unclear | Unclear | Yes | Yes | Not applicable | Not applicable | Yes | Unclear |
| Belet, (2018) Belgium | Reducing interethnic bias through real-life and literary encounters: The interplay between face-to-face and vicarious contact in high school classrooms | Yes | Yes | Yes | Yes | Yes | Yes | Yes | Unclear |
| Bikmen and Sunar, (2013), Turkey | Difficult dialogs: Majority group membersâ€™ willingness to talk about inequality with different minority groups | No | No | Yes | Yes | Yes | Yes | Yes | Unclear |
| Bohman and Miklikowska, (2021), Sweden | Does classroom diversity improve intergroup relations? Short- and long-term effects of classroom diversity for cross-ethnic friendships and anti-immigrant attitudes in adolescence | Unclear | Yes | Yes | Yes | Not applicable | Not applicable | Yes | Yes |
| Brenick and Romano, (2016), United States | Perceptions of discrimination by young diaspora migrants: Individual- and school-level associations among adolescent ethnic German immigrants | Yes | Yes | Yes | Yes | Yes | Yes | Yes | Unclear |
| Brenick et al., (2012), Germany | Perceived Peer and Parent Out-Group Norms, Cultural Identity, and Adolescents' Reasoning About Peer Intergroup Exclusion | Yes | No | Yes | Yes | Unclear | Unclear | Yes | Unclear |
| Celebi et al., (2014), Turkey | Out-group trust and conflict understandings: The perspective of Turks and Kurds in Turkey | No | Yes | Yes | Yes | Yes | Yes | Yes | No |
| Chavez et al., (2021), Chile | The role of perspective-taking and low social class prejudice on cross-ethnic friendship formation. | Yes | Yes | Yes | Yes | Yes | Yes | Yes | Unclear |
| Gönültaş and Mulvey, (2023b), Turkey | Does negative media representation shape adolescents' discrimination towards Syrian refugees through threat perception and prejudice? | No | No | Yes | Yes | Yes | Yes | Yes | No |
| Gönültaş and Mulvey, (2022), Turkey | Theory of Mind as a Correlate of Bystanders' Reasoning About Intergroup Bullying of Syrian Refugee Youth | Yes | Yes | Yes | Yes | Yes | Yes | Yes | Unclear |
| Gönültaş and Mulvey, (2023a), Turkey | Do Adolescents Intervene in Intergroup Bias-based Bullying? Bystander Judgments and Responses to Intergroup Bias-based Bullying of Refugees | Yes | Yes | Yes | Yes | Yes | Yes | Yes | Unclear |
| Hitti and killen, (2015), United States | Expectations About Ethnic Peer Group Inclusivity: The Role of Shared Interests, Group Norms, and Stereotypes. | Unclear | No | Yes | Yes | Yes | Yes | Yes | Unclear |
| Hitti and killen, (2023), United States | Adolescents' evaluations of those who challenge exclusive and inclusive peer norms | Yes | Unclear | Yes | Yes | No | No | Yes | No |
| Hitti et al., (2023), United States | What motivates adolescent bystanders to intervene when immigrant youth are bullied? | No | No | Yes | Yes | Yes | Yes | Yes | Yes |
| Hooijsma and Juvonen, (2021), Netherlands | Two sides of social integration: Effects of exposure and friendships on second- and third-generation immigrant as well as majority youth's intergroup attitudes | Yes | Yes | Yes | Yes | Yes | Yes | Yes | Unclear |
| Jumageldinov, (2014), Kazakhstan | Ethnic Identification, Social Discrimination and Interethnic Relations in Kazakhstan | Unclear | Unclear | Unclear | Yes | Not applicable | Not applicable | Yes | Unclear |
| Kaufmann, (2021), Canada | Integrating canadian youth: The state of intergroup contact, belonging, and support for immigration | No | No | No | Yes | Not applicable | Not applicable | Yes | No |
| Kisfalusi et al., (2020), Hungary | Bullying and victimization among majority and minority students: The role of peersâ€™ ethnic perceptions | No | Unclear | Yes | Yes | Not applicable | Not applicable | Yes | Unclear |
| Kretschmer and Leszczensky, (2022), Germany | In-Group Bias or Out-Group Reluctance? The Interplay of Gender and Religion in Creating Religious Friendship Segregation among Muslim Youth | Unclear | Yes | Yes | Yes | Not applicable | Not applicable | Yes | Yes |
| Lintner et al., (2023), Czech Republic | Ukrainian refugees struggling to integrate into Czech school social networks | No | Unclear | No | Yes | Not applicable | Not applicable | Yes | Not applicable |
| Maor and Gross, (2023), Israel | The moderating effects of group dominance and religiosity on the relationship between social rejection during school years and attitudes toward minorities in adulthood | No | Not applicable | No | Yes | Not applicable | Not applicable | Yes | Not applicable |
| Munayer and Horenczyk, (2014), Israel | Multi-group acculturation orientations in a changing context: Palestinian Christian Arab adolescents in Israel after the lost decade. | No | No | Yes | Yes | Not applicable | Not applicable | Yes | No |
| Palmer et al., (2023), Berlin | Challenging the exclusion of immigrant peers | No | Unclear | Yes | Yes | Not applicable | Not applicable | Yes | Not applicable |
| Plenty and Jonsson, (2017), Sweden | Social Exclusion among Peers: The Role of Immigrant Status and Classroom Immigrant Density. | Yes | Yes | Yes | Yes | Not applicable | Not applicable | Yes | Yes |
| Rienties and Nolan, (2014), United Kingdom | Understanding friendship and learning networks of international and host students using longitudinal Social Network Analysis | Yes | Yes | Yes | Yes | Yes | Yes | Yes | Yes |
| Smith and Minescu, (2021), Ireland | Comparing normative influence from multiple groups: Beyond family, religious ingroup norms predict childrenâ€™s prejudice towards refugees | Yes | Yes | Yes | Yes | Yes | Yes | Yes | Unclear |
| Spiegler et al., (2024), England; the Netherlands; Germany; Sweden | Classroom ethnic diversity, teacher support, and peer victimization: Evidence from four European countries | Yes | Unclear | Yes | Yes | Yes | Yes | Yes | Yes |
| Stark et al., (2015), Netherlands | Liking and disliking minority-group classmates: Explaining the mixed findings for the influence of ethnic classroom composition on interethnic attitudes | Yes | Yes | Yes | Yes | Yes | Yes | Yes | Unclear |
| Szabo et al., (2020), Hungary | Social contact configurations of international students at school and outside of school: Implications for acculturation orientations and psychological adjustment | Unclear | Unclear | Unclear | Yes | Yes | Yes | Yes | Unclear |
| Wang et al., (2020), China | Positive and negative intergroup contact and willingness to engage in intergroup interactions among majority (Han) and minority (Uyghur) group members in China: The moderating role of social dominance orientation | Yes | No | Yes | Yes | Yes | Yes | Yes | No |
| Yüksel et al., (2022), Britain | When do bystanders get help from teachers or friends? Age and group membership matter when indirectly challenging social exclusion | Unclear | Unclear | Yes | Yes | Yes | Yes | Yes | Yes |
| Zhou et al., (2022), United States | Do cross-race friendships with majority and minority peers protect against the effects of discrimination on school belonging and depressive symptoms? | Yes | No | No | Yes | Yes | Yes | Yes | Unclear |
| Jumageldinov, (2014), Kazakhstan | Ethnic Identification, Social Discrimination and Interethnic Relations in Kazakhstan | Unclear | Unclear | Unclear | Yes | Not applicable | Not applicable | Yes | Unclear |
